# Understanding modulations of lipid mediators in cancer using a murine model of carcinomatous peritonitis

**DOI:** 10.1002/cam4.4699

**Published:** 2022-03-22

**Authors:** Makoto Kurano, Eri Sakai, Yutaka Yatomi

**Affiliations:** ^1^ Department of Clinical Laboratory Medicine The University of Tokyo Tokyo Japan; ^2^ Department of Clinical Laboratory The University of Tokyo Hospital Tokyo Japan

**Keywords:** cancer, eicosanoids, liquid chromatography‐mass spectrometry, lysophospholipids, sphingolipids

## Abstract

**Background:**

Numerous studies have investigated the possible involvement of eicosanoids, lysophospholipids, and sphingolipids in cancer. We considered that comprehensive measurement of these lipid mediators might provide a better understanding of their involvement in the pathogenesis of cancer. In the present study, we attempted to elucidate the modulations of sphingolipids, lysophospholipids, diacyl‐phospholipids, eicosanoids, and related mediators in cancer by measuring their levels simultaneously by a liquid chromatography‐mass spectrometry method in a mouse model of carcinomatous peritonitis.

**Methods:**

We investigated the modulations of these lipids in both ascitic fluid and plasma specimens obtained from Balb/c mice injected intraperitoneally with Colon‐26 cells, as well as the modulations of the lipid contents in the cancer cells obtained from the tumor xenografts.

**Results:**

The results were as follows: the levels of sphingosine 1‐phosphate were increased, while those of lysophosphatidic acid (LysoPA), especially unsaturated long‐chain LysoPA, tended to be increased, in the ascitic fluid. Our findings suggested that ceramides, sphingomyelin, and phosphatidylcholine, their precursors, were supplied by both de novo synthesis and from elsewhere in the body. The levels of lysophosphatidylserine (LysoPS), lysophosphatidylinositol, lysophosphatidylglycerol, and lysophosphatidylethanolamine were also increased in the ascitic fluid, while those of phosphatidylserine (PS), a precursor of LysoPS, were markedly decreased. The levels of arachidonic acid derivatives, especially PGE2‐related metabolites, were increased, while the plasma levels of eicosanoids and related mediators were decreased. Comprehensive statistical analyses mainly identified PS in the ascitic fluid and eicosanoids in the plasma as having highly negative predictive values for cancer.

**Conclusions:**

The results proposed many unknown associations of lipid mediators with cancer, underscoring the need for further studies. In particular, the PS/LysoPS pathway could be a novel therapeutic target, and plasma eicosanoids could be useful biomarkers for cancer.

## Graphical abstract

Up to the present, numerous studies have investigated the possible involvement of lipid mediators, including eicosanoids, lysophospholipids, and sphingolipids, in cancer., however few studies have conducted comprehensive measurements of these lipid mediators simultaneously, although these lipid mediators have the same origins, to some degree. In this study, simultaneously investigated the modulations of these lipids in both ascitic fluid and plasma specimens obtained from Balb/c mice injected intraperitoneally with Colon‐26 cells. Comprehensive statistical analyses mainly identified phosphatidylserine in the ascitic fluid and eicosanoids in the plasma as having highly negative predictive values for cancer, suggesting that the phosphatidylserine/lysophosphatidylserine pathway could be a novel therapeutic target, and plasma eicosanoids could be useful biomarkers for cancer.

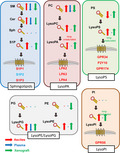

## 1 INTRODUCTION

It remains an important task to elucidate the pathogenesis of cancer at present, because of the substantial unmet needs, especially in regard to the development of effective medications. Among the candidate molecules, both basic and clinical studies have demonstrated the association of lipid mediators with cancer. Since specific G‐protein coupled receptors (GPCRs) have been identified for several lipid mediators, these are also deemed as potential therapeutic targets; in particular, some agonists and antagonists of these GPCRs have been identified as promising novel candidates in the field of oncotherapeutics.

Eicosanoids, which are derived from arachidonic acid (AA), have been reported to promote the progression of cancer^1^, ^2^; especially, the association between prostaglandin E2 (PGE2) and colon cancer is well‐known. Both basic and clinical studies have shown that inhibition of PGE2 production by COX inhibitors suppresses colon cancer.^3^, ^4^ Dietary EPA and DHA supplementation has been demonstrated to suppress cancer in human subjects^5^, ^6^, ^7^ and the metabolites derived from these ω3 fatty acids have been demonstrated to exhibit anti‐cancer activity through suppression of inflammation and/or the induction of apoptosis.^8^

Lysophospholipids are also classified as lipid mediators, just like eicosanoids and other derivatives of ω3 fatty acids, and specific GPCRs for several lysophospholipids have been identified.^9^ Among lysophospholipids, the associations of lysophosphatidic acids (LysoPA, LPA) and sphingosine 1‐phosphate (S1P) with cancer have been well studied. LysoPA is known to induce cancer cell proliferation, invasion, and migration,^10^, ^11^ and also to regulate tumor immunity.^12^ S1P has also been shown to exert potent anti‐apoptotic activity and promote cancer progression.^13^, ^14^ In addition, emerging important physiological roles of other lysophospholipids, such as lysophosphatidylserine (LysoPS, LPS) and lysophosphatidylinositol (LysoPI, LPI)^15^, ^16^ suggests the possible involvement of these lipids in the development of human diseases, including cancer.^17^, ^18^ Moreover, ceramides and sphingosine (Sph), a precursor of S1P, have been shown to possess potent anti‐apoptotic activity and been proposed as having the capability for suppressing cancer progression.^19^ The ceramide‐S1P rheostat theory has been proposed, which hypothesizes that the balance between ceramides and S1P might determine the fate of cancer cells.^20^

Although a large number of basic and clinical studies have been conducted to investigate the involvement of these lipid mediators in cancer, few studies have conducted comprehensive measurements of these lipid mediators simultaneously. These lipid mediators have the same origins, to some degree. For example, diacyl‐phospholipids undergo hydrolysis into fatty acids, and lysophospholipids and eicosanoids and derivatives of ω3 fatty acids can be produced from these fatty acids.^21^ In regard to sphingolipids, ceramides, Sph, and S1P, as well as that of sphingomyelin (SM), which is a precursor of ceramides, and dihydrosphingosine (dhSph) and dihydrosphingosine 1‐phosphate (dhS1P), which possess similar structures and biological activities to Sph and S1P, respectively, are all close in the metabolic pathway.^22^ Moreover, recent studies proposed a possible crosstalk between receptors for specific lipid mediators represented by LPA1 and S1P1.^23^, ^24^ Therefore, a comprehensive understanding of the modulations of these bioactive lipids is necessary to elucidate the roles of the lipid mediators in the pathogenesis of cancer.

In the present study, based on the above background, we attempted to elucidate the modulations of lipid mediators and related lipids, including sphingolipids, lysophospholipids, diacyl‐phospholipids, eicosanoids and related mediators, by measuring their levels simultaneously in murine models of carcinomatous peritonitis using a liquid chromatography‐mass spectrometry (LC–MS/MS) method. We investigated the modulations of these lipids in both the ascitic fluid, which would reflect an environment akin to that of cancer, and plasma, together with their modulations within the cancer cells collected from the tumor xenografts in order to obtain a better understanding of the involvement of these lipids in the pathogenesis of cancer; we also considered the possible usefulness of the mediators as laboratory biomarkers and therapeutic targets.

## 2 MATERIALS AND METHODS

### 2.1 Cell experiments

Colon‐26 cells were directly obtained from the Cell Resource Center for Biomedical Research, Tohoku University (TKG 0518). They were cultured in RPMI1640 medium (R8758; Sigma‐Aldrich Co.) containing 10% fetal bovine serum (FBS; 554‐02655; BioSera Inc.) and 1% penicillin/streptomycin (168–23,191; WAKO Pure Chemical Industries).

### 2.2 Animal experiments

Six‐week‐old male Balb/c mice, obtained from Japan SLC (Shizuoka, Japan), were injected intraperitoneally with Colon‐26 cells at 2 × 10^6^/0.1 ml PBS. We prepared the 2‐deoxyglucose probe by dissolving IRDye 800CW 2‐DG (LI‐COR Biosciences) in PBS at 0.1 nmol/μl. On day 8 after the administration, the animals were injected with 10 nmol of a 2‐deoxyglucose probe or an equal volume of PBS via the tail vein.^25^ After a further 48 h, the animals were anesthetized by intraperitoneal injection of sodium pentobarbital (Somnopentyl; Kyoritsu Seiyaku Co.) at the dose of 40 mg/kg body weight, followed by collection of plasma and ascitic fluid samples. Ascitic fluid samples were collected by injection of 2 ml of PBS into the peritoneal space, followed by retrieval of the fluid after 3 min. Then, the mice were euthanized by cervical dislocation before they emerged from the anesthesia, and intraperitoneal samples, including gastrointestinal tract (stomach to colon), spleen, and pancreas, were collected.

To compare the lipid contents and mRNA levels of the receptors for lipid mediators, we seeded a six‐well plate with Colon‐26 cells. Then, Colon‐26 cells from some of the wells were intraperitoneally injected into Balb/c mice as described above, while those from other wells were collected for the above analyses. Cancerous tissue specimens were also collected from the peritoneal xenografts for further analyses.

All the animal experiments were conducted in accordance with the guidelines for animal care and with the approval of the Animal Care Committee of the University of Tokyo (P17‐076).

### 2.3 Evaluation of the dissemination of intraperitoneally injected Colon‐26 cells

After washing twice with PBS, the signals and areas of the injected 2‐deoxyglucose probe in the collected intraperitoneal organs were evaluated using the Odyssey CLx Infrared Imaging System (LI‐COR Biosciences) in accordance with the manufacturer's protocol.

### 2.4 Measurement of S1P, ceramides and sphingosine, glycero‐lysophospholipids, diacyl‐phospholipids, and eicosanoids and related mediators

We measured the levels of the lipid mediators listed below by five independent LC–MS/MS methods using an LC8060 system consisting of a quantum ultra‐triple quadrupole mass spectrometer (Shimadzu, Japan). We simultaneously measured six ceramide species (Cer d18:1/16:0 [C16:0], Cer d18:1/18:0 [C18:0], Cer d18:1/18:1 [C18:1], Cer d18:1/20:0 [C20:0], Cer d18:1/22:0 [C22:0], Cer d18:1/24:0 [C24:0]), Sph, and dhSph, as previously described.^26^ We also measured S1P and dhS1P as described previously.^27^ Furthermore, LysoPA, lysophosphatidylcholine (LysoPC, LPC), LysoPS, LysoPI, lysophosphatidylglycerol (LysoPG, LPG), and lysophosphatidylethanolamine (LysoPE, LPE) were also measured, as described previously.^28^, ^29^ We monitored 12 acyl chains (14:0, 16:0, 16:1, 18:0, 18:1, 18:2, 18:3, 20:3, 20:4, 20:5, 22:5, and 22:6) in the present study. We also measured 155 eicosanoids and related mediators, described in Table S1, together with AA, EPA, and DHA, as described previously,^30^ in addition to SM and diacyl‐phospholipids, including phosphatidylcholine (PC), phosphatidylethanolamine (PE), phosphatidylinositol (PI), phosphatidylglycerol (PG), and phosphatidylserine (PS).^30^, ^31^ We monitored 17 diacyl chains (32:1, 32:2, 34:1, 34:2, 36:1, 36:2, 36:3, 36:4, 38:1, 38:2, 38:3, 38:4, 38:5, 38:6, 40:1, 40:2, 40:7) for SM and 64 diacyl chains (28:0, 28:1, 28:2, 30:0, 30:1, 30:2, 32:0, 32:1, 32:2, 32:3, 32:4, 34:0, 34:1, 34:2, 34:3, 34:4, 34:5, 34:6, 36:0, 36:1, 36:2, 36:3, 36:4, 36:5, 36:6, 36:7, 38:0, 38:1, 38:2, 38:3, 38:4, 38:5, 38:6, 38:7, 38:8, 40:0, 40:1, 40:2, 40:3, 40:4, 40:5, 40:6, 40:7, 40:8, 40:9, 40:10, 42:0, 42:1, 42:2,42:3, 42:4, 42:5, 42:5, 42:6, 42:7, 42:8, 42:9, 42:10, 42:11, 44:0, 44:1, 44:2, 44:6, 44:7, 44:12) for PC, PE, PI, PG, and PS. For these methods except that for SM and diacyl‐phospholipids, both the intra‐day and inter‐day coefficients of variation for almost all metabolites are below 20%, as validated in our previous papers.^26^, ^27^, ^28^, ^31^

### 2.5 Reverse‐transcriptase PCR

The total RNA extracted from the murine tissues or cells using the GenElute Mammalian Total RNA Miniprep kit was subjected to reverse transcription with the ReverTra Ace qPCR RT Master Mix. Quantitative PCR was performed using an ABI 7300 Real‐Time PCR System (Applied Biosystems), with the primers for the S1P receptor constructed in a previous paper^32^ and commercially available primers listed in Table S2. The expression levels of the genes of interest were normalized to those of the endogenous control GAPDH mRNA.

### 2.6 Statistical analysis

The results are expressed as dot plots. The data were analyzed using SPSS or SIMCA (MKS Umetrics). Differences between two groups were evaluated using the Mann–Whitney U test and correlations between two parameters were evaluated by Spearman's correlation test. *p* values of <0.05 were deemed as being indicative of statistical significance in all the analyses. The orthogonal projection to latent structures (OPLS) was statistically analyzed using SIMCA to explore the variables associated with cancer. The independent effects of the lipid metabolites in the ascitic fluid and plasma on the progression or dissemination of cancer or body weight were evaluated using a stepwise multiple regression analysis.

## 3 RESULTS

### 3.1 Validation of the murine models of carcinomatous peritonitis (Colon‐26 mice)

First, we validated the animal models used. We observed the tumor xenografts in all of the eight mice injected with Colon‐26 cells (Figure S1A,B). When the spread of the cancerous cells was evaluated with an imaging analyzer, the signals and are of accumulated 2‐deoxyglucose probe were higher in the mice injected with Colon‐26 (Colon‐26 mice) than in those injected with PBS (control mice) (Figure S1C–F). The protein levels in the collected ascitic fluid specimens from the Colon‐26 mice were also higher, while body weights were not significantly different between the PBS and Colon‐26 mice (Figure S1G,H). These findings served to validate the murine models of carcinomatous peritonitis used in this study.

### 3.2 Modulations of sphingolipid levels in the ascitic fluid in the murine models of carcinomatous peritonitis (Colon‐26 mice)

**FIGURE 1:**
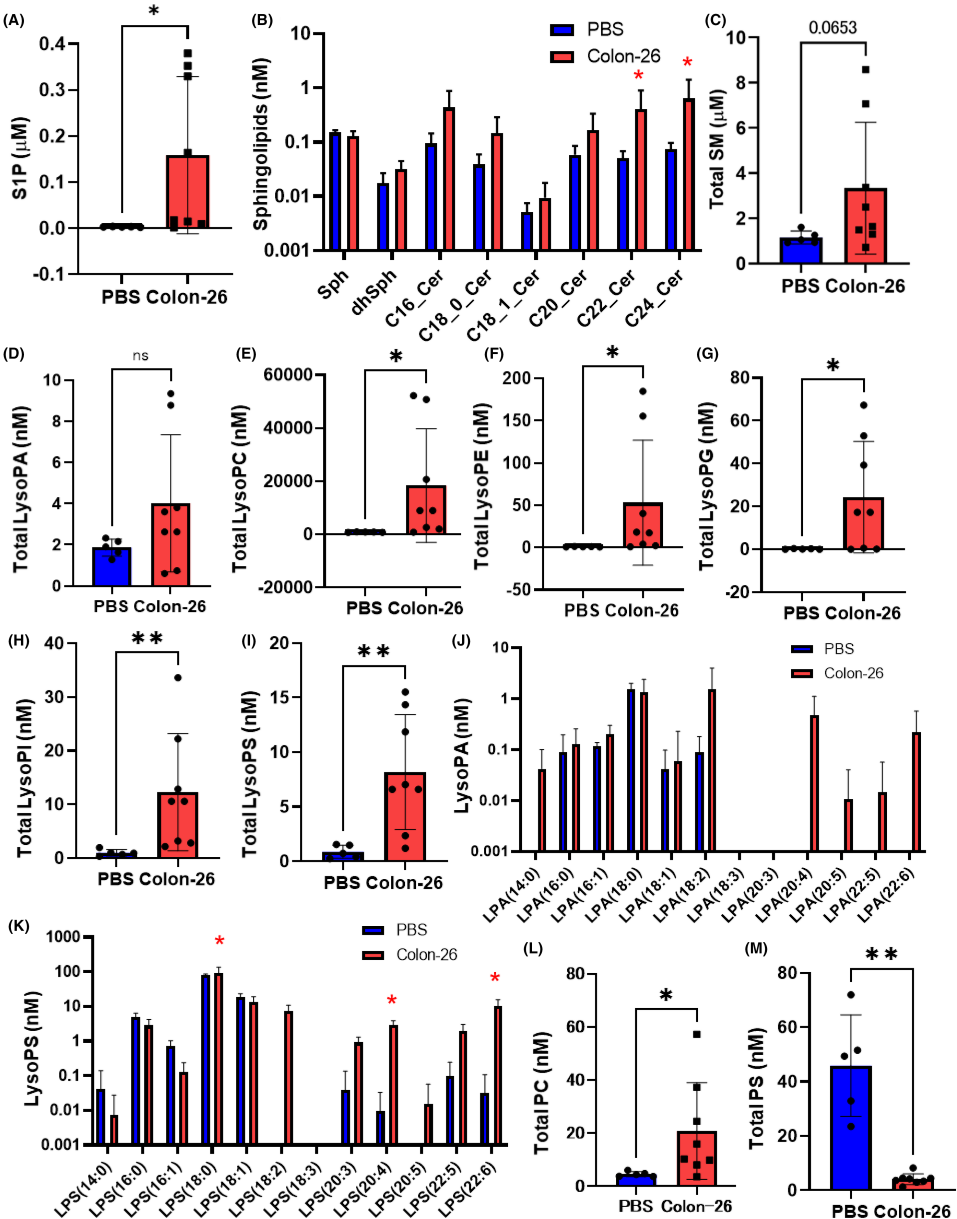
Modulations of sphingolipids, glycerol‐lysophospholipids, and diacyl‐phospholipids in the ascitic fluid in murine models of carcinomatous peritonitis (Colon‐26 mice). Ascitic fluid specimens of mice injected intraperitoneally with Colon‐26 cells (Colon‐26 mice, *n* = 8) or PBS (PBS mice, *n* = 5) were collected as described in the *Materials and Methods* section, for the measurement of sphingolipids, glycerol‐lysophospholipids, and diacyl‐phospholipids by a LC–MS/MS method. (A) S1P, (B) sphingosines and ceramides, (C) total SM, (D) total LysoPA, (E) total LysoPC, (F) total LysoPE, (G) total LysoPG, (H) total LysoPI, (I) total LysoPS, (J) LysoPA species, (K) LysoPS species, (L) total PC, and (M) total PS. Differences were evaluated using the Mann–Whitney *U* test. *p < 0.05, **p < 0.01. The boxes represent the means of independent samples, and the bars represent the SD

As shown in Figure 1A,B and Figure S2A, the levels of S1P, C22:0 Cer, and C24:0 Cer were significantly higher, and those of the other sphingolipids also tended to be higher, in the ascitic fluid of the Colon‐26 mice. Although the differences in the total SM levels did not reach statistical significance, the levels of several species of SM were higher in the specimens of the Colon‐26 mice (Figure 1C and Figure S2B).

### 3.3 Modulations of glycerophospholipids in the ascitic fluid in the murine models of carcinomatous peritonitis (Colon‐26 mice)

Next, we investigated the modulations of the glycero‐lysophospholipids and diacyl‐phospholipids. The total levels of all the lysophospholipids, except LysoPA, in the ascitic fluid of the Colon‐26 mice were significantly higher (Figure 1D–I). The levels of LysoPA species, especially long‐chain unsaturated species, also tended to be higher, although the difference did not reach statistical significance (Figure 1J). In regard to the species of lysophospholipids other than LysoPA, large differences in the levels of almost all species of LysoPC and LysoPE were observed, (Figure S3A,B), while the differences were observed in the levels of specific species of LysoPS, LysoPG, and LysoPI (Figure 1K, Figure S3C,D). In regard to the diacyl‐phospholipids, the total PC levels were higher, whereas the total PS levels were lower in the ascitic fluid of the Colon‐26 mice (Figure 1L,M, Figure S4A–C). The levels of PC and PS species were higher and lower, respectively, in the Colon‐26 mice (Figure S4D,E).

### 3.4 Modulations of eicosanoids and related mediators in the murine models of carcinomatous peritonitis (Colon‐26 mice)

**FIGURE 2:**
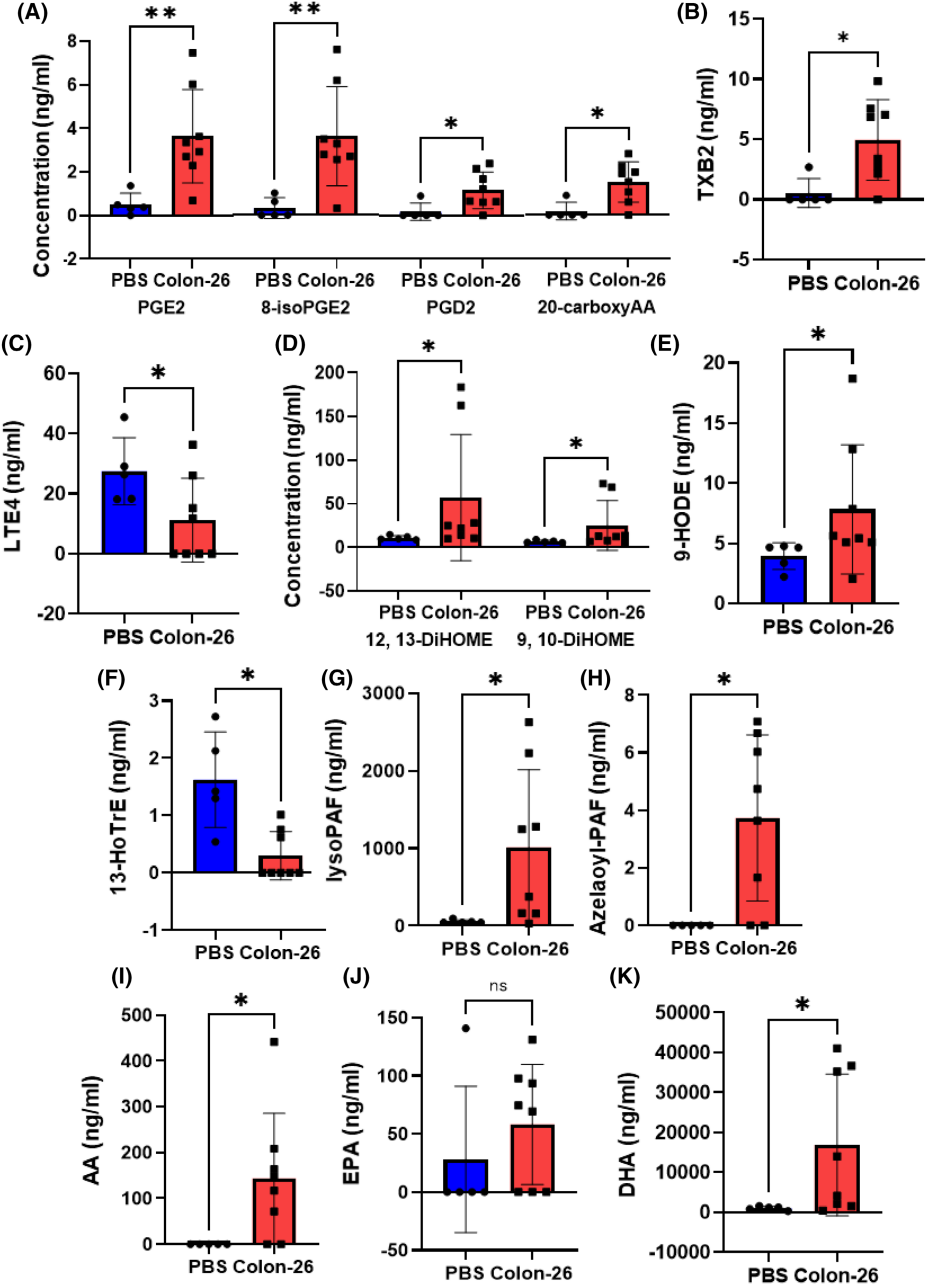
Modulations of eicosanoids and related mediators in the ascitic fluid in murine models of carcinomatous peritonitis (Colon‐26 mice). Ascitic fluid specimens of mice injected intraperitoneally with Colon‐26 cells (Colon‐26 mice, *n* = 8) or PBS (PBS mice, *n* = 5) were collected as described in the *Materials and Methods* section for the measurements of sphingolipids, glycerol‐lysophospholipids, and diacyl‐phospholipids by a LC–MS/MS method. (A–C) derivatives of arachidonic acid (AA), (D, E) derivatives of linoleic acid, (F) derivatives of α‐linolenic acid, (G, H) PAF‐related metabolites, and (I–K) AA, EPA, and DHA. Differences were evaluated using the Mann–Whitney *U* test. *p < 0.05, **p < 0.01. The boxes represent the means of independent samples, and the bars represent the SD

In regard to eicosanoids and related mediators, the levels of several mediators derived from AA were higher in the ascitic fluid specimens of the Colon‐26 mice, including PGE2, 8‐isoPGE2, PGD2, 20‐carboxy AA, and TXB2; only the LTE4 levels were lower (Figure 2A–C). The levels of 12,13‐DiHoME and 9,10‐DiHOME, and 9‐HODE, which are linoleic acid‐derived metabolites, were higher (Figure 2D,E). The levels of 13‐HOTrE, which is derived from α‐linolenic acid, were lower, while those of lyso‐PAF and azelaoyl‐PAF were also higher (Figure 2F–H). Elevation in the levels of the AA metabolites was accompanied by elevation of the level of AA (Figure 2I). Interestingly, although the levels of DHA and EPA were significantly higher or tended to be higher in the ascitic fluid specimens of the Colon‐26 mice (Figure 2J,K), we observed no differences in the ascitic fluid levels of DHA and EPA‐derived mediators between the Colon‐26 and control mice.

### 3.5 Modulations of sphingolipids and glycerophospholipids in the plasma of the murine models of carcinomatous peritonitis (Colon‐26 mice)

**FIGURE 3:**
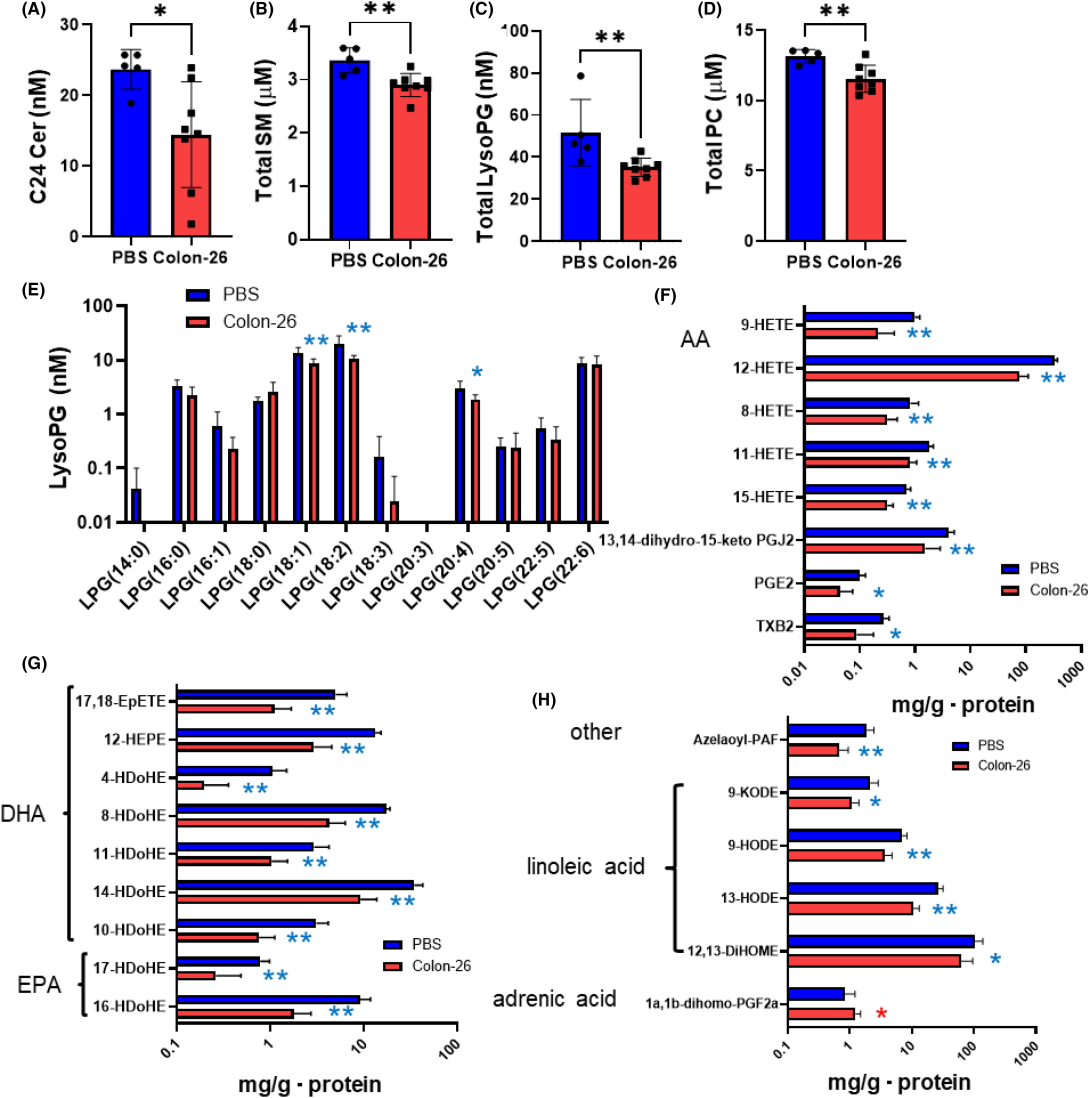
Modulations of the lipid mediators in the plasma in murine models of carcinomatous peritonitis (Colon‐26 mice). Plasma samples of mice injected intraperitoneally with Colon‐26 cells (Colon‐26 mice, *n* = 8) or PBS (PBS mice, *n* = 5) were collected as described in the *Materials and Methods* section for the measurement of lipid mediators by a LC–MS/MS. (A) C24:0 ceramide, (B) total SM, (C) total LysoPG, (D) total PC, (E) LysoPG species, (F) derivatives of arachidonic acid (AA), (G) DHA and EPA derivatives, (H) other eicosanoids related mediators. Differences were evaluated using the Mann–Whitney *U* test. *p < 0.05, **p < 0.01. The boxes represent the mean of independent samples, and the bars represent SD

We also investigated the modulations of lipids in the plasma of the Colon‐26 mice. Interestingly, in comparison to the modulations in the ascitic fluid, no remarkable modulations were observed in the plasma levels of sphingolipids and glycerophospholipids. For the case of the sphingolipids, only the levels of C24 Cer and SM were significantly lower in the Colon‐26 mice (Figure 3A,B, and Figure S5A–D). As for glycero‐lysophospholipids, only the LysoPG levels were lower in the Colon‐26 mice (Figure 3C,E, Figure S5E–I). In regard to diacyl‐phospholipids, only the total PC levels were significantly lower in the Colon‐26 mice (Figure 3D, Figure S5J–M). The total PS levels also tended to be lower, and the levels of several species of plasma PS were significantly lower in the Colon‐26 mice (Figure S5N).

### 3.6 Modulations of eicosanoids and related mediators in the plasma in the murine models of carcinomatous peritonitis (Colon‐26 mice)

In contrast to the non‐remarkable changes in the plasma levels of sphingolipids and glycerophospholipids, more obvious modulations were observed for eicosanoids and related mediators in the plasma than in the ascitic fluid, although the plasma levels of AA, EPA, and DHA were not significantly modulated (Figure S6). The levels of several species of lipid mediators derived from AA, EPA, and DHA were significantly lower in the Colon‐26 mice (Figure 3F,G). Levels of metabolites derived from linoleic acid and azelaoyl‐PAF were also lower, while the levels of 1a,1b‐dihomo‐PGF2a, which is derived from adrenic acid, were higher in the Colon‐26 mice (Figure 3H).

### 3.7 The levels of LysoPS and PE in the ascitic fluid had significant correlations with those in the plasma in the murine models of carcinomatous peritonitis (Colon‐26 mice)

When we investigated the correlations between the levels of lipid metabolites in the ascitic fluid and those in the plasma, we observed positive significant correlations between the ascites concentration and the plasma concentration in C16:0 Cer, C18:0 Cer, 14:0 and 20:4 LysoPE, 36:0, 42:9, and 42:10 PC, 36:1, 36:2, 36:4, 38:3, 38:4, 38:5, 38:6, 40:5, 40:6, 40:7, 40:8, and 40:9 PE, 15‐keto‐PGF2a, 5,6‐DHET, and OEA, while negative ones in 14:0 and 18:3 LysoPC, 16:0 LysoPG, 18:1, 18:2, 20:4, and 22:6 LysoPS, 38:0, 38:8, and 44:6 PC, and 38:2 PI (Table S3). Especially, we observe negative correlations in the concentrations of LysoPS and positive correlations in those of PE. Anyway, since we observed no significant correlations for many metabolites, the lipid metabolites in the ascitic fluid and in the plasma might be determined by different factors which are involved in the pathogenesis of cancer.

### 3.8 Comprehensive OPLS analyses for the lipids in the plasma or ascitic fluid related to carcinomatous peritonitis

Next, we collated all the results for the lipid mediators together and conducted statistical analysis by the OPLS method to explore the variables in the ascitic fluid or plasma that might be associated with carcinomatous peritonitis. For the ascitic fluid, the score plot, loading scatter plot, and loading column plot for the OPLS are shown in Figure S7. The lipids in the ascitic fluid with positive and negative predictive values for cancerous peritonitis with higher significant variable importance in projection are listed in Table 1A. Relatively short‐chain PE (32:0 PE, 32:1 PE), long polyunsaturated PC (42:6 PC, 38:8 PC), and 18:0 LysoPS in the ascitic fluid were selected as having a high positive predictive values for carcinomatous peritonitis, just like PGE2 and related metabolites, while various species of PS in the ascitic fluid were selected as having a highly negative predictive values for carcinomatous peritonitis. For the plasma, the score plot, loading scatter plot, and loading column plot for the OPLS are shown in Figure S8. As shown in Table 1B, 36:1 SM, 34:1 SM, 36:5 PI, 40:5 PC, and 44:12 PC, together with tetranor‐PGEM, and 15‐deoxy‐delta‐12,14‐PGJ2, which are derived from AA, and 1a,1b‐dihomo‐PGF2a, which is derived from adrenic acid, were selected as metabolites with positive predictive values for cancer. Various eicosanoids and related mediators were selected as having negative predictive values, including AA derivatives such as 12‐HETE, 11‐HETE, 9‐HETE, and 15 HETE, DHA derivatives such as 8‐HDHA, 14‐HDoHE, 16‐HDHA and 10‐HDHA, EPA derivatives such as 12‐HEPE and 17,18‐EpETE, and linoleic acid derivatives such as 13‐HODE, and lyso‐PAF.

**TABLE 1:** OPLS analysis with variable importance in projection for cancerous peritonitis

Variables with positive predictive values		Variables with negative predictive values	
Variables	VIP	Variables	VIP
(A) Ascitic fluid			
PE(32:0)	0.114405 ± 0.104641	PS(40:5)	−0.115413 ± 0.1072160
PE(32:1)	0.105762 ± 0.0692964	PS(38:2)	−0.113551 ± 0.0695944
PC(42:6)	0.0999738 ± 0.0266533	PS(38:8)	−0.107826 ± 0.0674715
LPS(18:0)	0.0993638 ± 0.0528489	PS(34:1)	−0.107728 ± 0.0802837
PC(38:8)	0.0983716 ± 0.0616061	PS(38:4)	−0.104599 ± 0.0779812
PGE2	0.0903447 ± 0.0866163	PS(36:0)	−0.102331 ± 0.0880706
8‐iso‐PGE2	0.0903087 ± 0.0804461	13‐HOTrE	−0.0995477 ± 0.0924346
20‐carboxy‐AA	0.0886722 ± 0.038243	PS(38:1)	−0.0821211 ± 0.0810363
SM(40:7)	0.0882251 ± 0.0341509		
Azelaoyl‐PAF	0.0853918 ± 0.0384171		
TXB2	0.0848424 ± 0.0375254		
PC(32:2)	0.0835664 ± 0.0379516		
(B) Plasma			
SM(36:1)	0.0780367 ± 0.0576787	12‐HETE	−0.126589 ± 0.039955
tetranor‐PGEM	0.077347 ± 0.0447706	8‐HDHA	−0.125803 ± 0.052404
15‐deoxy‐delta‐12,14‐ PGJ2	0.0733176 ± 0.0615282	12‐HEPE	−0.124223 ± 0.0353725
1a,1b‐dihomo‐PGF2a	0.0665987 ± 0.0580077	13‐HODE	−0.121628 ± 0.0338489
SM(34:1)	0.0650266 ± 0.0502934	14‐HDoHE	−0.11905 ± 0.0550225
PI(36:5)	0.0627294 ± 0.050669	16‐HDHA	−0.117545 ± 0.0510917
PC(40:5)	0.0546072 ± 0.054417	17,18‐EpETE	−0.117145 ± 0.0430533
PC(44:12)	0.0501912 ± 0.0353491	11‐HETE	−0.116075 ± 0.0761063
		9‐HETE	−0.11418 ± 0.038879
		15‐HETE	−0.113037 ± 0.0760719
		10‐HDHA	−0.112496 ± 0.0580481
		Azelaoyl‐PAF	−0.111358 ± 0.0454642

*Note*: Data are means ± 2.40419 × SE. VIP means variable importance in projection, which was calculated by OPLS analyses using SIMCA.

### 3.9 Comprehensive multiple regression analyses to identify the lipids in the ascitic fluid and plasma related to the progression of carcinomatous peritonitis

We also performed multiple regression analyses with the signal intensity of cancer as an objective variable to identify the lipid metabolites in the ascitic fluid or plasma related to the progression of cancer. In the ascitic fluid, 44:2 PC, 13‐HpODE, 12‐KETE, 8‐iso‐13,14‐dihydro‐15‐keto‐PGF2a, and 34:4 PC were identified as positive explanatory variables and EPA as a negative one. In the plasma, 38:1 PE, 12‐HEPE, PGA2, and 20:3 LPI were selected as positive explanatory variables, and 34:2 PC and 36:6 PE as negative ones.

### 3.10 Comprehensive multiple regression analyses to identify the lipids in the ascitic fluid and plasma related to body weight loss

The body weight might decrease when cancer progresses, while in the duration of the present experiment, some Colon‐26 mice lost their body weight, while others did not (Figure S1H). We also performed multiple regression analyses with body weight as an objective variable to identify the lipid metabolites in the ascitic fluid or plasma related to body weight loss. In the ascitic fluid, 16:0 LysoPA, 38:5 PS, and C22:0 Cer were identified as positive explanatory variables and 32:0 PE, 22:6 LysoPA, and 13‐HpODE as negative ones (Table S4A). In the plasma, 12‐HEPE, 20‐carboxy‐AA, 15‐HEPE, and EPA were selected as positive explanatory variables, and 11,12‐DHET and 15‐HETE as negative ones (Table S4B).

### 3.11 Modulations of the expression levels of receptors for lipid mediators and the lipid contents within the Colon‐26 tumor xenograft tissue

**FIGURE 4:**
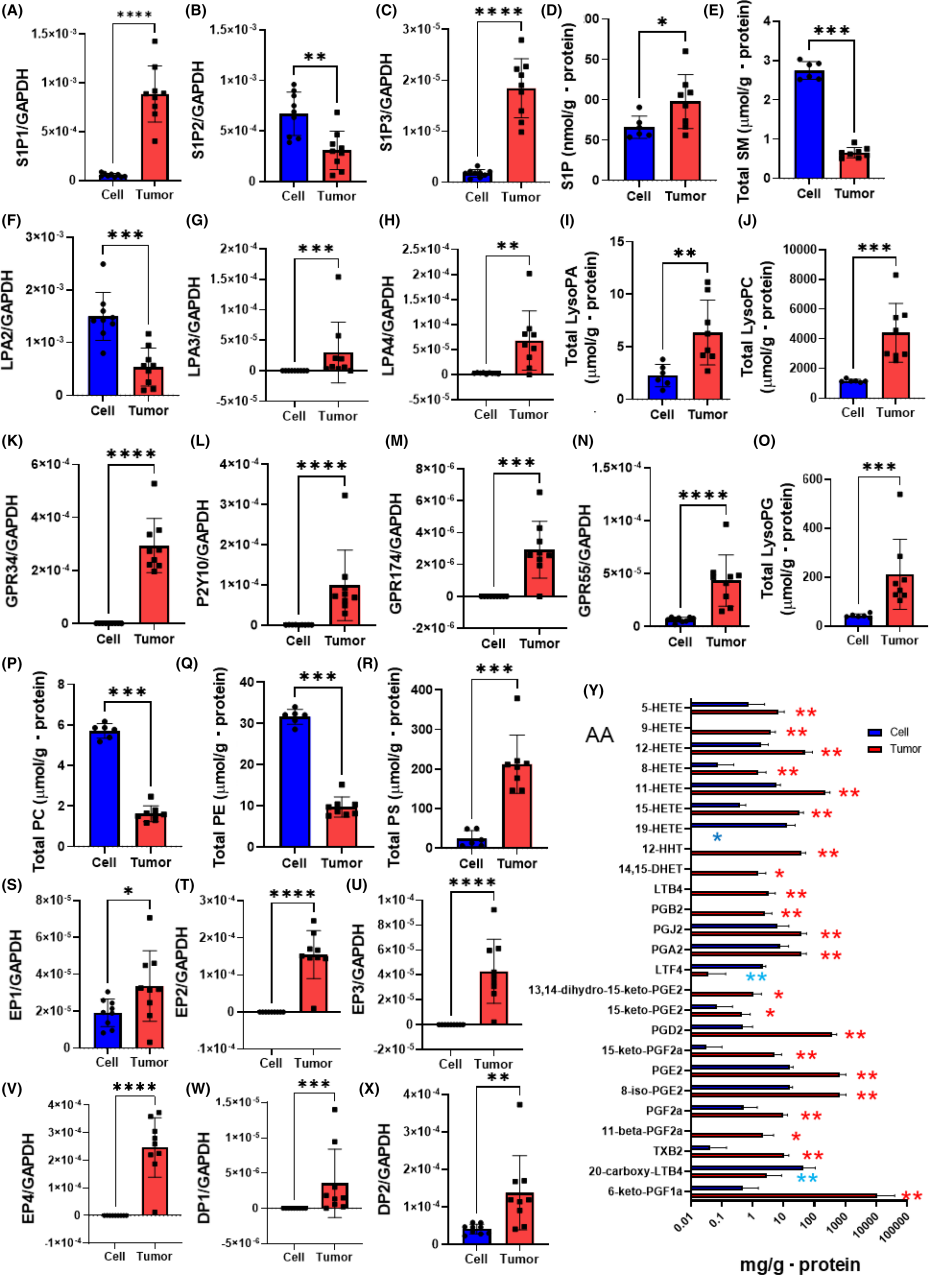
Modulation of the expression of receptors for lipid mediators and the levels of lipid mediators within the Colon‐26 tumor xenograft tissue. Colon‐26 cells were seeded on to six‐well plates at the same time, and Colon‐26 cells from some wells were intraperitoneally injected into Balb/c mice, as illustrated in Figures 1, 2, 3, and those from other wells were collected for the analyses (*n* = 6). Specimens of cancerous tissue from the abdominal tumor xenografts were also collected for further analyses (*n* = 8). The mRNA expression levels of the receptors for the lipid mediators were determined by a real‐time PCR method using GAPDH as the internal standard. The lipid contents were determined by LC–MS/MS. (A–C) mRNA levels of the S1P receptors, (D) S1P, (E) total SM, (F–H) mRNA levels of LPA receptors, (I) LysoPA, (J) LysoPC, (K–M) mRNA levels of LysoPS receptors, (N) mRNA levels of GPR55, (O) LysoPG, (P) total PC, (Q) total PE, (R) and total PS, (S–X) mRNA expression levels of the prostaglandin receptors, (Y) and derivatives of arachidonic acid (AA). Differences were evaluated using the Mann–Whitney *U* test. *p < 0.05, **p < 0.01, ***p < 0.001, ****p < 0.0001. The boxes represent the means of independent samples, and the bars represent the SD

Next, we investigated the modulations of the expression levels of receptors for lipid mediators and the lipid contents within the Colon‐26 tumor xenograft tissue. While the expression of S1P1 and S1P3 increased, that of S1P2 decreased after the engraftment (Figure 4A–C). The contents of S1P and dhS1P, together with those of their precursors, dhSph and C16:0, C20:0, C22:0, C24:0 Cer, were increased, while those of C18:0 Cer and SM were decreased within the Colon‐26 tumor xenograft tissue (Figure 4D,E, Figure S9A,B). In regard to LysoPA, the expressions of LPA2, LPA3, and LPA4 increased, just like the levels of LysoPA and its precursor LysoPC (Figure 4F–J and Figure S9C–E). For LysoPS, the expression of GPR34, P2Y10, and GPR174 increased (Figure 4K–M). Although there was no modulation of the total LysoPS levels, the levels of 16:0 LysoPS and 16:1 LysoPS levels were slightly decreased, while those of 18:2 LysoPS, 20:3 LysoPS, 20:4 LysoPS, 22:5 LysoPS, and 22:6 LysoPS greatly increased within the Colon‐26 tumor xenograft tissue (Figure S9F,G). For LysoPI and LysoPG, the expression of GPR55 increased and the levels of LysoPG increased within the Colon‐26 tumor xenograft tissue (Figure 4N,O). Although the total LysoPI levels were not modulated, the levels of 16:0 LysoPI, 16:1 LysoPI, and 18:1 LysoPI were decreased, while those of 18:2 LysoPI, 20:4 LysoPI, 22:5 LysoPI, and 22:6 LysoPI levels were increased (Figure S9H,I). We also observed that the contents of LysoPE increased within the Colon‐26 tumor xenograft tissue (Figure S9J). In regard to the precursors of the glycero‐lysophospholipids, the levels of PC and PE were increased, whereas those of PS were decreased, and those of PI and PG remained unchanged within the Colon‐26 tumor xenograft tissue (Figure 4P–R and Figure S9K,L).

When we investigated the modulation of the receptors for eicosanoids, we observed that the expression levels of all of the monitored receptors for eicosanoids were increased within the Colon‐26 tumor xenograft tissue (Figure 4S–X and Figure S10A–C). In regard to the levels of eicosanoids and related mediators, the metabolites whose levels were significantly modulated are shown in Figure 4Y and Figure S10D,E. Levels of the derivatives of EPA, DHA, ethanolamide, dihomo‐γ‐linolenic acid, α‐linolenic acid, and adrenic acid were, in general, increased within the Colon‐26 tumor xenograft tissue (Figure S10D,E). Levels of most of the derivatives of AA were increased, while levels of 19‐HETE, LTF4, and 20‐carboxy LTB4 levels were decreased (Figure 4Y). Among the derivatives of linoleic acid, levels of 9‐KODE, 13‐KODE and 9‐HpODE levels were decreased, whereas those of 9‐HODE and 13‐HODE were increased within the Colon‐26 tumor xenograft tissue (Figure S10E). The levels of AA, EPA, and DHA were also increased within the Colon‐26 tumor xenograft tissue (Figure S10F–H).

## 4 DISCUSSION

**FIGURE 5:**
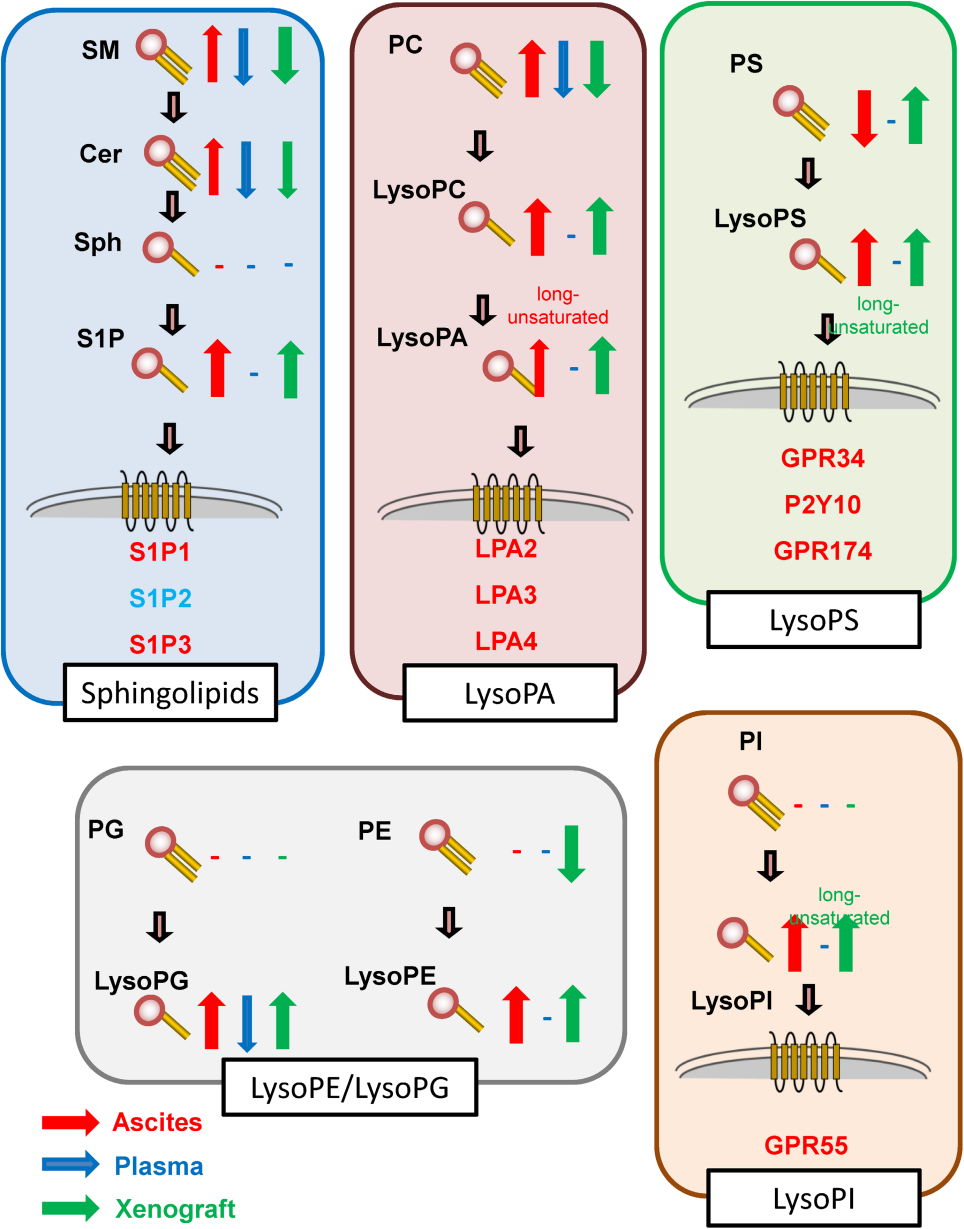
Summary of the modulations of sphingolipids and lysophospholipids. Red and blue arrows represent the modulations in ascitic fluid and plasma specimens obtained from murine models of carcinomatous peritonitis, respectively. Green arrows represent the modulations within the Colon‐26 tumor xenograft tissue

**FIGURE 6:**
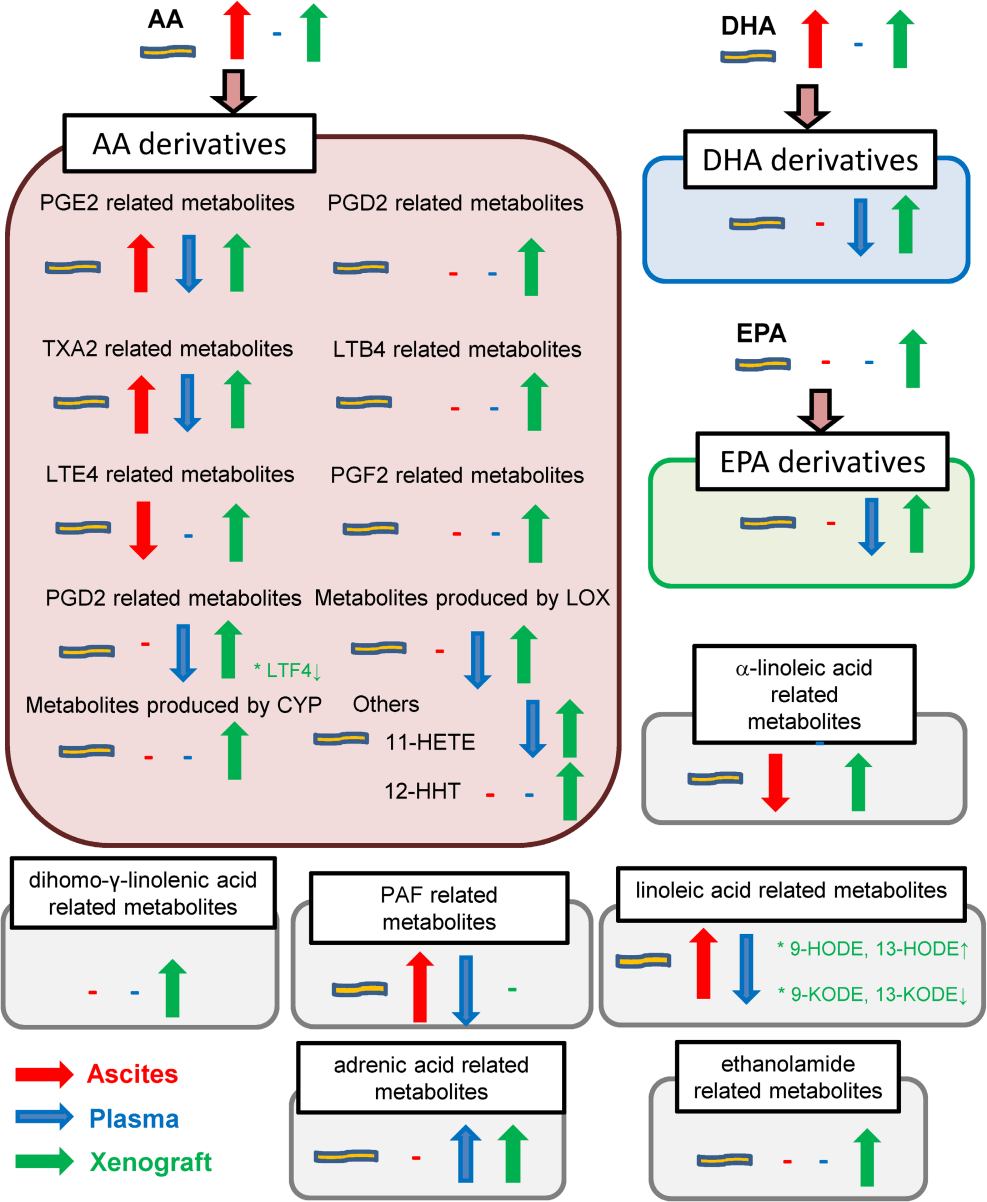
Summary of the modulations of eicosanoids and related mediators. Red and blue arrows represent the modulations in ascitic fluid and plasma specimens obtained from murine models of carcinomatous peritonitis, respectively. Green arrows represent the modulations within the Colon‐26 tumor xenograft tissue

In the present study, we simultaneously measured the levels of lipid mediators and related lipids, including sphingolipids, lysophospholipids, diacyl‐phospholipids, eicosanoids and related mediators in the ascitic fluid and plasma of murine models of carcinomatous peritonitis, together with the modulation of these lipids within the Colon‐26 tumor xenograft tissue to comprehensively understand the modulations and potential roles of the lipid mediators. We have summarized the results in Figures 5 and 6.

In regard to the sphingolipids, the levels of S1P were increased in the ascitic fluid and within the Colon‐26 tumor xenograft tissue, concordant with the potential roles of S1P in the progression of cancer suggested by basic studies.^19^, ^20^ The modulations of ceramides and SM, precursors of S1P, were, however, different; their levels were increased in the ascitic fluid but decreased within the Colon‐26 tumor xenograft tissue. The S1P‐ceramide rheostat theory might be a valid theory for cancer cells, while in vivo, SM and ceramides might be supplied from elsewhere in the body due to the increased capillary permeability usually observed in advanced cancers.^33^ Actually, the plasma levels of SM and ceramides were decreased in our murine models. Among the ubiquitously expressed S1P receptors, the expression levels of S1P1 and S1P3 were increased, while those of S1P2 were decreased. In human subjects, the levels of all of S1P1, S1P2, and S1P3 levels have been reported to be higher in patients with colon cancer,^34^, ^35^, ^36^ and the levels of S1P to be higher in patients with breast cancer^37^ and cancerous meningitis.^27^ In general, the activation of S1P1 and S1P3 induces cell proliferation mainly through the PI3K/AKT pathway, while that of S1P2 induces a contractile response through the Rho/Rock pathway.^38^ At present, the role of S1P2 in the progression of cancer remains controversial.^39^, ^40^

In regard to LysoPA, although the difference did not reach statistical significance, the levels of unsaturated long‐chain LysoPA tended to be increased in the ascitic fluid, accompanied by significant elevation of the levels of LysoPC and PC, whereas the levels of LysoPA and LysoPC were increased and those of PC were decreased within the Colon‐26 tumor xenograft tissue. These results suggest enhanced conversion of PC to LysoPC, followed by elevation of LysoPA levels in cancer cells, and PC might be supplied from elsewhere in the body as SM and ceramide. In fact, the plasma levels of PC were decreased. Among the LPA receptors, we observed elevation in the expressions of LPA2, LPA3, and LPA4 within the Colon‐26 tumor xenograft tissue. Considering that unsaturated long‐chain LysoPA, levels of which were especially increased in the ascitic fluid, possesses potent agonist activity for LPA3,^41^ the LPA3 signal might be significantly activated in murine carcinomatous peritonitis. In previous studies, activation of LPA1, LPA2, and LPA3 enhanced the proliferation and motility of cancer cells,^10^ while activation of LPA4 promoted angiogenesis.^42^ In human subjects, upregulation of LPA2 and downregulation of LPA1 has been demonstrated in colon cancer,^43^ and upregulation of LPA1 and LPA3 and association of LPA2 and LPA6 expression with differentiation has been demonstrated in hepatocellular carcinoma.^44^, ^45^ The level of LysoPA in the ascitic fluid has also been reported to be associated with the prognosis.^46^

Along with S1P and LysoPA, several classes of lysophospholipids are also emerging as potent bioactive lipid mediators. The levels of LysoPS were increased in the ascitic fluid, while those of PS, a precursor of LysoPS, were markedly decreased. The levels of both LysoPS, especially the unsaturated long‐chain form, and PS, together with the expression of three LysoPS receptors were significantly increased within the Colon‐26 tumor xenograft tissue. These results suggest that the production of LysoPS is accelerated in cancer cells, accompanied by enhanced production of PS, which would be insufficient to cover the demand of PS as precursors for LysoPS in cancer cells. The levels of LysoPI and LysoPG were also increased in the ascitic fluid. LysoPI is an agonist of GPR55.^47^ In humans, we have reported that the levels of LysoPS and LysoPI were higher in the ascitic fluid of patients with gastric cancer than in that of patients with cirrhosis^48^ or the cancer tissue in colorectal cancer.^49^ Expression of GPR34 in hepatocellular carcinoma and that of GPR55 in breast cancer have been reported to be positively correlated with the prognosis.^50^, ^51^ The role of LysoPS in the pathogenesis of cancer has remained unclear. LysoPS reportedly stimulates the migration of colorectal cancer cells^52^ and might suppress tumor immunity directly or indirectly.^12^ Although involvement of the LysoPI/GPR55 axis in the pathogenesis of cancer has been proposed,^18^ it has still not been established. In addition, we observed elevation of LysoPG and LysoPE in the ascitic fluid and within the Colon‐26 tumor xenograft tissue. Although the roles of these lysophospholipids in the pathogenesis of cancer remain unclear, elevation of their levels has been reported in human subjects with gastric cancer.^48^

As shown in Figure 3, marked modulations of AA derivatives were observed, especially elevated levels of PGE2‐related metabolites in the ascitic fluid together with increased expressions of the receptors for PGE2 within the Colon‐26 tumor xenograft tissue (Figure 4S–V), which is concordant with the established roles of PGE2 in colorectal cancer, as described in the *Introduction* section. Increased levels of TXA2, which is known to stimulate colonic cancer cell proliferation,^53^ in the ascitic fluid, as well as increased expression levels of TXA2r within the Colon‐26 tumor xenograft tissue were also observed. Increased levels of 12,13‐DiHoME and 9,10‐DiHOME and 9‐HODE, which are linoleic acid‐derived metabolites, and of PAF‐related metabolites were observed. Considering that these metabolites are involved in the progression of inflammation, these results might be rational.^54^, ^55^, ^56^, ^57^ On the contrary, the levels of LTE4 and 13‐HOTrE were decreased in the ascitic fluid. Modulation of 13‐HOTrE might contribute to the pathogenesis of cancer, since this metabolite possess anti‐inflammatory properties,^58^ while LTE4 has been proposed to be positively associated with inflammation and oxidation,^59^ which are well‐known mechanisms underlying the development of cancer.

Increases in the levels of most eicosanoids and related metabolites within the Colon‐26 tumor xenograft tissue seemed reasonable, considering that these are involved in inflammation. However, it is noteworthy that the plasma levels of eicosanoids and related mediators were decreased, without any changes of the plasma AA, EPA, and DHA levels. These results were concordant with a previous study conducted using the serological samples of human subjects with colorectal cancer,^60^ while partially contradicting previous reports indicating that the levels of AA derivatives were higher, while those of linoleic acid derivatives were lower in serum samples collected from subjects with colorectal cancer.^61^ Considering that the levels of eicosanoids and related mediators are substantially influenced by platelet activation, we believe that their levels would decrease in circulation, although the precise mechanisms remain unclear. Based on the results of the present study, we propose the necessity to consider the involvement of eicosanoids and related mediators at the local site of a tumor and in distant sites separately. Actually, few metabolites had significant positive correlations between their concentrations in the ascitic fluid and those in plasma (Table S3).

When we investigated the modulations of the expression levels of receptors for lipid mediators and the lipid contents within the Colon‐26 tumor xenograft tissue, various changes were observed, suggesting the activation of many signals of lipid mediators (Figure 4 and Figures S9 and S10). For sphingolipids, S1P‐S1P1 and S1P3 signal might be enhanced, which might facilitate the progression of cancer, since S1P‐S1P1 and S1P3 axis accelerates cell proliferation.^38^ Although the upregulation of almost all ceramide species might be interpreted as anti‐cancer modulation, considering the pro‐apoptotic properties of ceramides,^19^ since SM levels decreased, these modulations might reflect the facilitated production of S1P from its precursor sphingolipids. For glycero‐phospholipids, LysoPA‐LPA3 and LPA4 axis, LysoPS‐GPR34, P2Y10, and GPR174 axis, and LysoPI or LygoPG‐GPR55 axis seemed to be activated. Although the roles of LysoPS in cancer remained unknown, the activation of LysoPA‐LPA3 and LPA4 and LysoPI or LysoPG‐GPR55 axis might contribute to the progression of cancer.^10^, ^18^, ^42^ For eicosanoids and related mediators, the signals of AA derivatives might be theoretically enhanced, which are especially concordant with the established roles of PGE2 pathway in the pathogenesis of colon cancer.^1^, ^2^ On the contrary, increased levels of EPA and DHA metabolites might serve as anti‐cancer properties.^8^ Considering their protective roles in inflammation, these modulations might be compensatory changes provoked against cancer cells in the mice. Anyway, most of the modulations of the receptors for lipid mediators and the lipid contents within the Colon‐26 tumor xenograft tissue seemed to accelerate the progression of cancer.

Lastly, we comprehensively analyzed the modulations of all the metabolites monitored in the present study to understand the involvement of these lipids in the pathogenesis of cancer. As shown in Table 1A, PE and PC, as well as PGE2‐related metabolites, were determined as metabolites with positive predictive values for cancer, while the PS and 13‐HOTrE were selected as metabolites with negative predictive values. Considering the established roles of PGE2 in cancer and the anti‐inflammatory properties of 13‐HOTrE,^58^ the associations of these eicosanoids with cancer seems reasonable. However, the association of diacyl‐phospholipids, especially PS, seemed interesting; modulation of the PS/LysoPS axis, especially the mechanisms of uptake of PS into cancer cells from their circumstances, might be a novel therapeutic target for cancer. As shown in Table 1B, the negative associations with cancer of eicosanoids and related metabolites is remarkable, suggesting that these mediators are promising as biomarkers for cancers, as proposed in the previous studies. In regard to the metabolites contributing to tumor progression, PC, 13‐HpODE, 12‐KETE, and 8‐iso‐13,14‐dihydro‐15‐keto‐PGF2a showed positive associations, while and EPA in the ascitic fluid showed negative associations. The observed associations of PC and EPA with tumor progression appears reasonable, since PC, levels of which are increased in cancer, is used as a component of the cell membrane^62^ and EPA has been shown to possess anti‐cancer properties.^8^ Although its role in cancer is less established, 13‐HpODE might promote cancer through phosphorylation of the EGF receptor.^63^ The physiological properties of 12‐KETE and 8‐iso‐13,14‐dihydro‐15‐keto‐PGF2a have not yet been elucidated. Regarding the metabolites related with the modulation of body weight in Colon‐26 mice, the selected metabolites were different from the metabolites selected for the progression of cancer. These results suggested the possible existence of different mechanisms, in which lipid mediators are involved, between cancer progression and body weight loss. Further experiments with longer observation are needed to address this point (Table 2).

**TABLE 2:** Multiple regression analysis to identify the metabolites associated with the degree of disseminations

	*B*	95% CI	Standardized β	*p* value
(A) Ascitic fluid				
PC(44:2)	2.37E+06	(2.35E+06–2.38E+06)	1.046	<0.001
13‐HpODE	1.90E+11	(1.90E+11–1.90E+11)	0.128	<0.001
12‐KETE	4.51E+06	(4.49E+06–4.52E+06)	0.125	<0.001
8‐iso‐13,14‐dihydro‐15‐keto‐PGF2a	2.17E+06	(2.16E+06–2.18E+06)	0.065	<0.001
PC(34:4)	3.38E+06	(3.35E+06–3.42E+06)	0.031	0.001
EPA	6.36E+08	(6.20E+08–6.52E+08)	−0.001	0.023
(B) Plasma				
PE(38:1)	3.65E+08	(3.65E+08–3.65E+08)	1.306	<0.001
PC(34:2)	−5.43E+07	(−5.43E+07 to −5.43E+07)	−0.360	<0.001
12‐HEPE	8.94E+05	(8.94E+05–8.94E+05)	0.053	<0.001
PGA2	9.29E+06	(9.29E+06–9.29E+06)	0.014	<0.001
PE(36:6)	−1.64E+06	(−1.64E+06 to −1.64E+06)	−0.001	<0.001
LPI(20:3)	−6.31E+02	(−6.37E+02 to −6.26E+02)	0.000	0.023

In the present study, we used a xenograft model with Colon‐26 cells, derived from colorectal cancer. Since we engrafted Colon‐26 cells into peritoneal cavity, this model might represent the peritoneal metastasis of colorectal cancer. However, in the respects of directly injecting cancer cells into peritoneal cavity, this model might represent the cancer originated from the peritoneum. Furthermore, the tumor tissues are composed of cancer cells and other cells such as immune cells. Therefore, we should consider the possibility that the modulation of lipid mediators might reflect the activation of immune cells as well as cancer cells. Among lipid metabolites we investigated in the present study, especially eicosanoids and related mediators are released from leukocytes. Further studies with human samples and those investigating tumor cells and immune cells in tumor tissues separately are needed to elucidate the roles of lipid mediators in the pathogenesis of cancer.

In summary, in the present study, we simultaneously analyzed the modulations of sphingolipids, lysophospholipids, diacyl‐phospholipids, eicosanoids, and related mediators in the ascitic fluid and plasma of murine models of carcinomatous peritonitis, together with their modulations within the Colon‐26 tumor xenograft tissue, and obtained the results summarized in Figure S5 and Figure 6. The results of the present study propose many unknown associations of lipid mediators with cancer and cancer progression, further elucidation of which warrants further basic and clinical studies. Especially, modulation of the PS/LysoPS pathway, especially the mechanisms of uptake of PS into cancer cells from their circumstances, might be a novel target in cancer therapeutics, and plasma eicosanoids and related mediators might serve as biomarkers of cancer.

## CONFLICT OF INTEREST

None declared.

## AUTHOR CONTRIBUTIONS

M. Kurano designed the research; M. Kurano analyzed the data; M. Kurano and E. Sakai performed the research; M. Kurano, K. and Y. Yatomi wrote the paper. All the authors have read and approve submission of this final manuscript.

## ETHICS APPROVAL AND CONSENT TO PARTICIPATE

All the animal experiments were conducted in accordance with the guidelines for animal care and with the approval of the Animal Care Committee of the University of Tokyo (P17‐076).

## Supporting information


**Appendix** S1

## Data Availability

All data generated or analyzed during this study are included in this article and its supplementary information files. The datasets generated or analyzed in the current study will be made available upon reasonable request.
