# Supplementary material for: Understanding modulations of lipid mediators in cancer using a murine model of carcinomatous peritonitis
Source: Cancer Med. 2022 Mar 22;11(18):3491–507. doi: 10.1002/cam4.4699 (PMC9487885; doi:10.1002/cam4.4699)
Supplement: Supplementary file 1 — Appendix S1 [file CAM4-11-3491-s001.pdf]

Supplemental Table S1. list of monitored eicosanoids and related mediators

Category	Compounds	Category	Compounds
AA	tetranor-PGFM	AA	LTF4
AA	tetranor-PGEM	AA	8-iso-PGA2
AA	tetranor-PGDM	AA	11-trans-LTC4
AA	20-hydroxy-PGF2a	AA	11-trans-LTE4
AA	20-hydroxy-PGE2	AA	PGA2
AA	18-carboxy-dinor-LTB4	AA	PGJ2
AA	13,14-dihydro-15-keto-tetranor-PGF1b	AA	PGB2
AA	2,3-dinor-8-iso-PGF2a	AA	8,12-iso-iPF2a-VI-1,5-lactone
AA	13,14-dihydro-15-keto-tetranor-PGF1a	AA	8,15-DiHETE
AA	6-keto-PGF1a	AA	6-trans-LTB4
AA	13,14-dihydro-15-keto-tetranor-PGD2	AA	5,15-DiHETE
AA	20-carboxy-LTB4	AA	LTB4
AA	20-hydroxy-LTB4	AA	13,14-dihydro-15-keto PGJ2
AA	13,14-dihydro-15-keto-tetranor-PGE2	AA	12-keto-LTB4
AA	6,15-diketo-13,14-dihydro-PGF1a	AA	N-acetyl-LTE4
AA	iPF2a-IV	AA	14,15-DHET
AA	8-iso-15(R)-PGF2a	AA	12-HHT
AA	8-iso-PGF2a	AA	11,12-DHET
AA	TXB2	AA	8,9-DHET
AA	11-beta-PGF2a	AA	20-carboxy-AA
AA	5-iPF2a-VI	AA	5,6-DHET
AA	8-iso-15-keto-PGF2a	AA	19-HETE
AA	PGF2a	AA	15-deoxy-delta-12,14-PGJ2
AA	8-iso-13,14-dihydro-15-keto-PGF2a	AA	20-HETE
AA	8-iso-PGE2	AA	18-HETE
AA	PGE2	AA	17-HETE

AA	11-dehydro-TXB2	AA	16-HETE
AA	15-keto-PGF2a	AA	15-HETE
AA	5S,14R-LXB4	AA	11-HETE
AA	PGK2	AA	8-HETE
AA	PGD2	AA	15-HpETE
AA	11-beta-13,14-dihydro-15-keto-PGF2a	AA	12-HETE
AA	15-keto-PGE2	AA	9-HETE
AA	14,15-LTC4	AA	5-HETE
AA	13,14-dihydro-15-keto-PGF2a	AA	12-HpETE
AA	5S,6R-LXA4	AA	12-KETE
AA	13,14-dihydro-15-keto-PGE2	AA	5,6-DHET-lactone
AA	LTD4	AA	5-HpETE
AA	5S,6S-LXA4	AA	14,15-EET
AA	14,15-LTE4	AA	5-KETE
AA	13,14-dihydro-15-keto-PGD2	AA	11,12-EET
AA	LTC4	AA	8,9-EET
AA	LTE4	AA	5,6-EET
DHA	Resolvin D2	DHA	13-HDoHE
DHA	Resolvin D1	DHA	10-HDoHE
DHA	Maresin1	DHA	14-HDoHE
DHA	10,17-DiHDoHE	DHA	11-HDoHE
DHA	7,17-hydroxy-DPA	DHA	7-HDoHE
DHA	20-HDoHE	DHA	8-HDoHE
DHA	16-HDoHE	DHA	4-HDoHE
DHA	17-HDoHE		
EPA	8-iso-PGF3a	EPA	18-HEPE
EPA	TXB3	EPA	15-HEPE
EPA	PGF3a	EPA	12-HEPE
EPA	PGE3	EPA	5-HEPE

EPA	PGD3	EPA	15-HpEPE
EPA	LXA5	EPA	12-HpEPE
EPA	17,18-DiHETE	EPA	5-HpEPE
EPA	14,15-DiHETE	EPA	17,18-EpETE
EPA	5,6-DiHETE		
ADA	1a1b-dihomo-PGF2a		
ALA	9-HOTrE	ALA	13-HOTrE
DGLA	TXB1	DGLA	PGD1
DGLA	8-iso-PGF1a	DGLA	8-iso-PGA1
DGLA	8-iso-PGE1	DGLA	PGA1
DGLA	PGE1	DGLA	15-HETrE
EA	PGF2a-EA	EA	11,12-EET-EA
EA	PGE2-EA	EA	8,9-EET-EA
EA	PGE1-EA	EA	5,6-EET-EA
EA	PGD2-EA	EA	AEA
EA	LTB4-EA	EA	OEA
EA	14,15-EET-EA		
EDA	15-HEDE	EDA	15-KEDE

Supplemental Table S2. The primers used for the quantitative PCR analyses

Gene name	Product number
mouse *LPA1*	Mm01346925_m1
mouse *LPA2*	Mm00469562_m1
mouse *LPA3*	Mm00469694_m1
mouse *LPA4*	Mm02620784_s1
mouse *LPA5*	Mm02621109_s1
mouse *LPA6*	Mm00613058_s1
mouse *GPR34*	Mm02620221_s1
mouse *P2Y10*	Mm02620706_s1
mouse *GPR174*	Mm01238430_m1
mouse *GPR55*	Mm02621622_s1
mouse *EP1*	Mm00443098_g1
mouse *EP2*	Mm00436051_m1
mouse *EP3*	Mm01316856_m1
human *EP4*	Mm00436053_m1
human *DP1*	Mm00436050_m1
human *DP2*	Mm00438315_s1
human *IP*	Mm00801939_m1
mouse *FP*	Mm00436055_m1
mouse *TXA2r*	Mm00436917_m1
mouse *Gapdh*	Mm99999915_g1

Supplemental Table S3. Correlations of the lipid metabolites between the ascites and the plasma in the murine models of carcinomatous peritonitis (Colon-26 mice)

metabolites	*r*	*p*-value	metabolites	*r*	*p*-value
C16:0 Cer	0.762	0.028	LPC(14:0)	-0.743	0.035
C18:0 Cer	0.738	0.037	LPC(20:3)	-0.743	0.035
			LPG(16:0)	-0.771	0.025
LPE(14:0)	1.000	0.000	LPS(18:1)	-0.724	0.042
LPE(20:5)	0.764	0.027	LPS(18:2)	-0.964	0.000
			LPS(20:4)	-0.898	0.002
PC(36:0)	0.833	0.010	LPS(22:6)	-0.761	0.028
PC(42:9)	0.723	0.043			
PC(42:10)	0.762	0.028	PC(38:0)	-0.830	0.011
PE(36:1)	0.833	0.010	PC(38:8)	-0.738	0.037
PE(36:2)	0.929	0.001	PC(44:6)	-0.837	0.010
PE(36:4)	0.830	0.011	PI(38:2)	-0.733	0.039
PE(38:3)	0.939	0.001			
PE(38:4)	0.905	0.002			
PE(38:5)	0.824	0.012			
PE(38:6)	0.738	0.037			
PE(40:5)	0.862	0.006			
PE(40:6)	0.929	0.001			
PE(40:7)	0.939	0.001			
PE(40:8)	0.862	0.006			
PE(40:9)	0.709	0.049			
15-keto-PGF2a	1.000	0.000			
5,6-DHET	0.781	0.022			
OEA	0.833	0.010			

Supplemental Table S4. Multiple regression analysis to identify the metabolites associated with body weight loss

A. Ascitic fluid

	B	95% CI			Standardized β	*P* value
LPA(16:0)	17.956	(17.901	- 18.011	)	1.110	<0.001
PE(32:0)	-35.714	(-36.059	- -35.372	)	-0.415	<0.001
LPI(22:6)	-15.447	(-15.543	- -15.351	)	-0.487	<0.001
13-HpODE	-0.156	(-0.163	- -0.150	)	-0.062	0.002
PS(38:5)	4.850	(4.575	- 5.124	)	0.077	0.003
C22:0 Cer	0.056	(0.042	- 0.069	)	0.013	0.012

B. Plasma

	B	95% CI				Standardized β	*P* value
12-HEPE	1.144	(1.144	-	1.144	)	.951	<0.001
11,12-DHET	-0.784	(-0.784	-	-0.784	)	-.527	<0.001
20-carboxy-AA	0.330	(0.330	-	0.330	)	.243	<0.001
15-HEPE	1.491	(1.490	-	1.491	)	.071	<0.001
EPA	2.322E-05	(2.305E-05	-	2.338E-05	)	.014	<0.001
15-HETE	-0.052	(-0.054	-	-0.050	)	-.002	0.002

Supplemental Figure S1

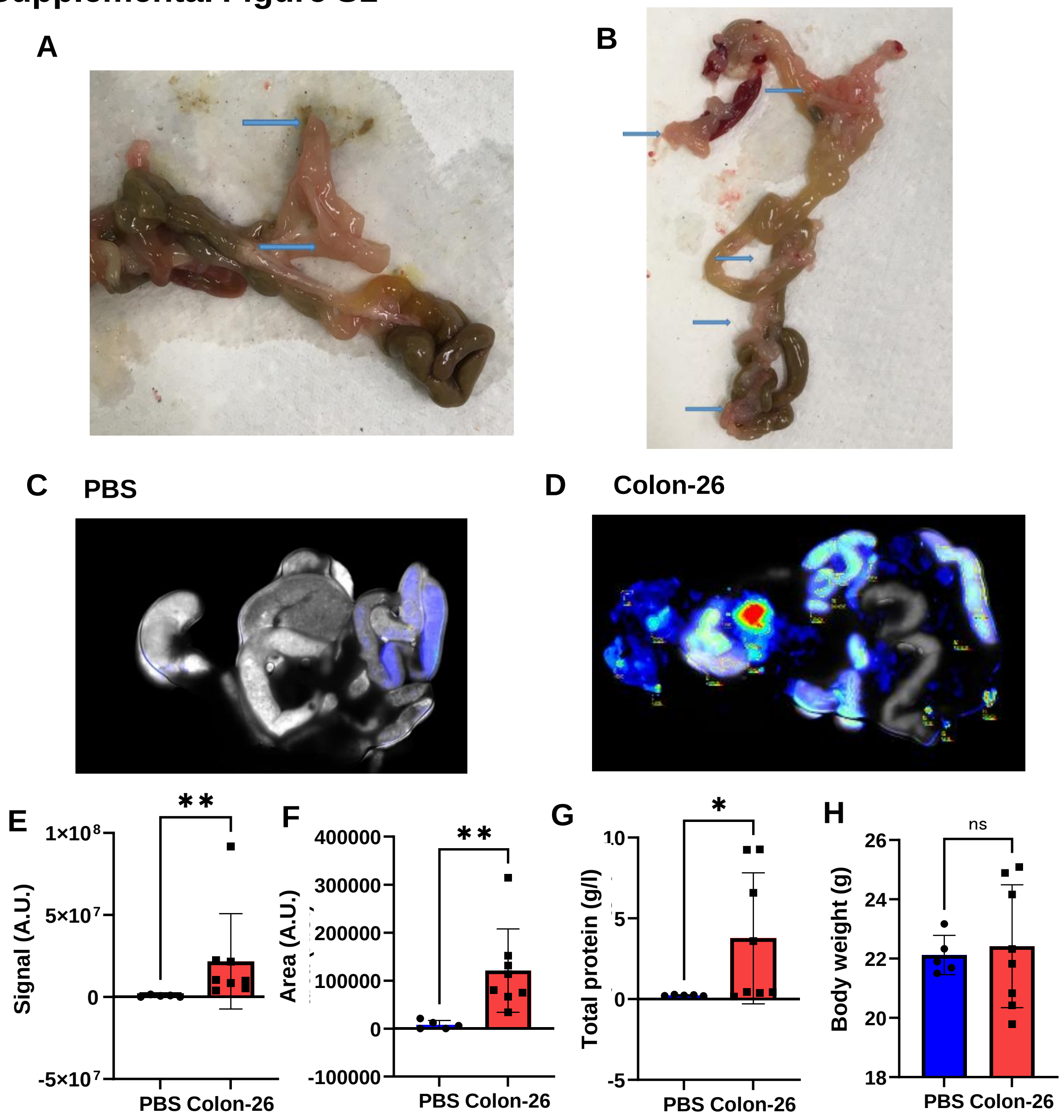

Supplemental Figure S1. Validation of the murine models of carcinomatous peritonitis Six-week-old Balb/c mice were injected intraperitoneally with Colon-26 cells (Colon-26, n = 8) or PBS (PBS, n = 5), as described in the *Materials and Methods* section. (A, B) Appearance of the disseminated tumors after engraftment. (C, D) Signal of 2-deoxyglucose in the intraperitoneal organs of the mice administered PBS (C) or Colon-26 cells (D). (E) Signal of 2-deoxyglucose. (F) Area of 2-deoxyglucose distribution. (G) Total protein levels in the ascitic fluid. (H) Body weight. Differences were evaluated using the Mann-Whitney U test. *$P < 0.05$, **$P < 0.01$. The boxes represent the means of independent samples, and the bars represent the S.D.

Supplemental Figure S2

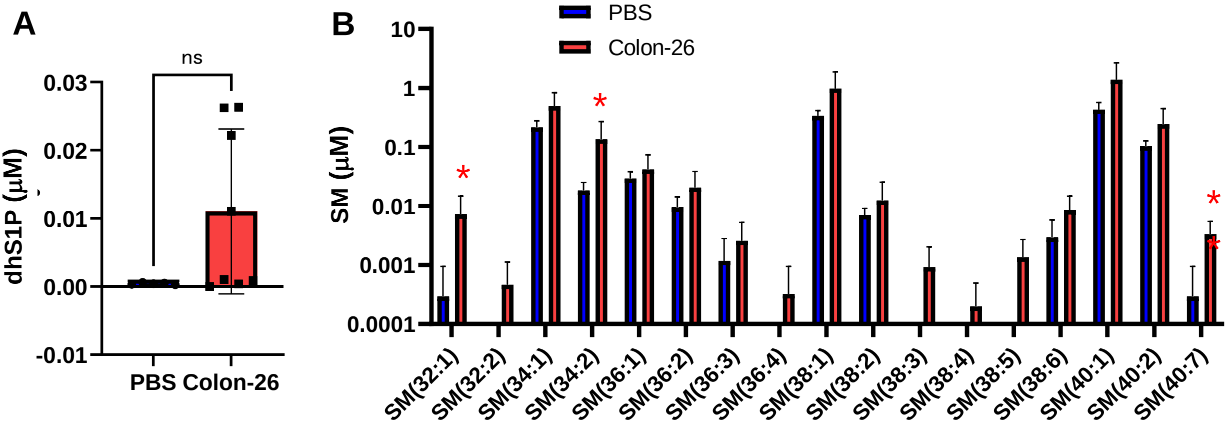

Supplemental Figure S2. Modulations of dhS1P and SM in the ascitic fluid of the murine models of carcinomatous peritonitis

Modulations of dihydrosphingosine 1-phosphate (dhS1P) (A) and SM species (B) in ascitic fluid, illustrated in Figure 1, are shown. Differences were evaluated using the Mann-Whitney U test. *P < 0.05, **P < 0.01. The boxes represent the means of independent samples, and the bars represent the S.D.

Supplemental Figure S3

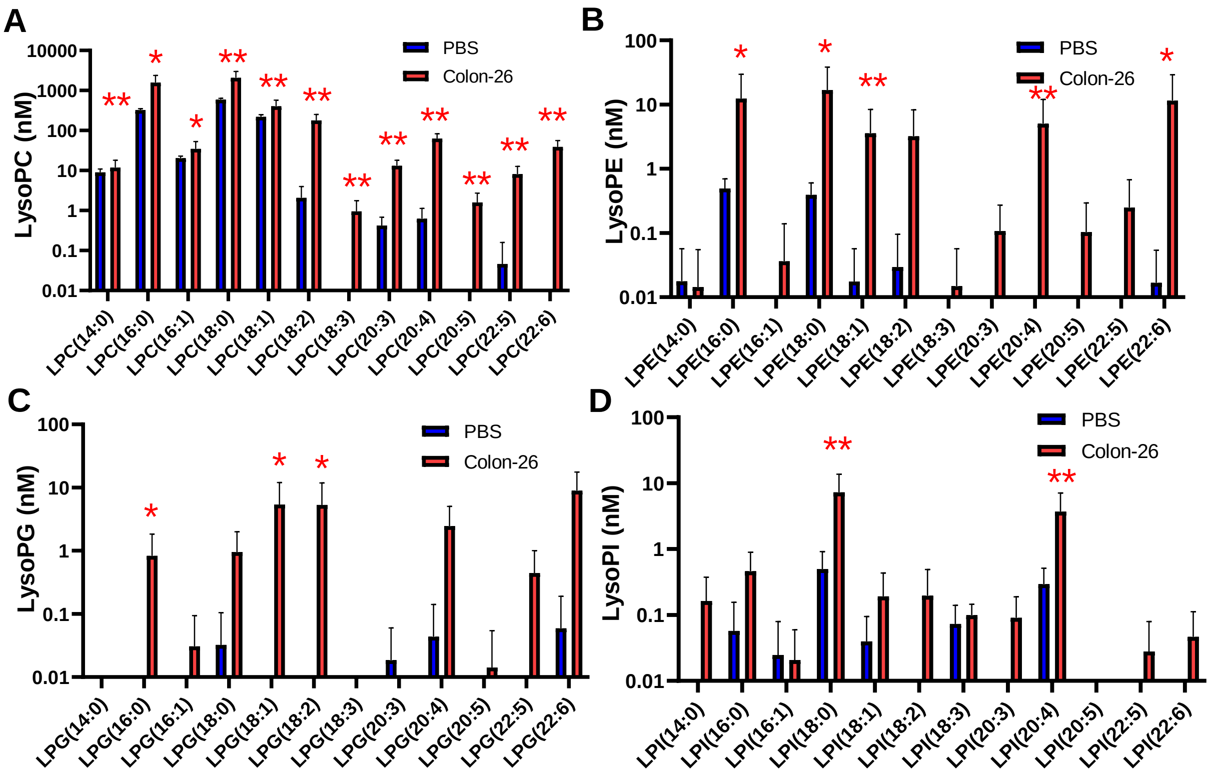

Supplemental Figure S3. Modulations of lysophospholipids in the ascitic fluid of murine models of carcinomatous peritonitis

Modulations of LysoPC species (A), LysoPE species (B), LysoPG species (C), and LysoPI species (D) in ascitic fluid, illustrated in Figure 1, are shown. Differences were evaluated using the Mann-Whitney U test. **P* < 0.05, ***P* < 0.01. The boxes represent the means of independent samples, and the bars represent the S.D.

Supplemental Figure S4

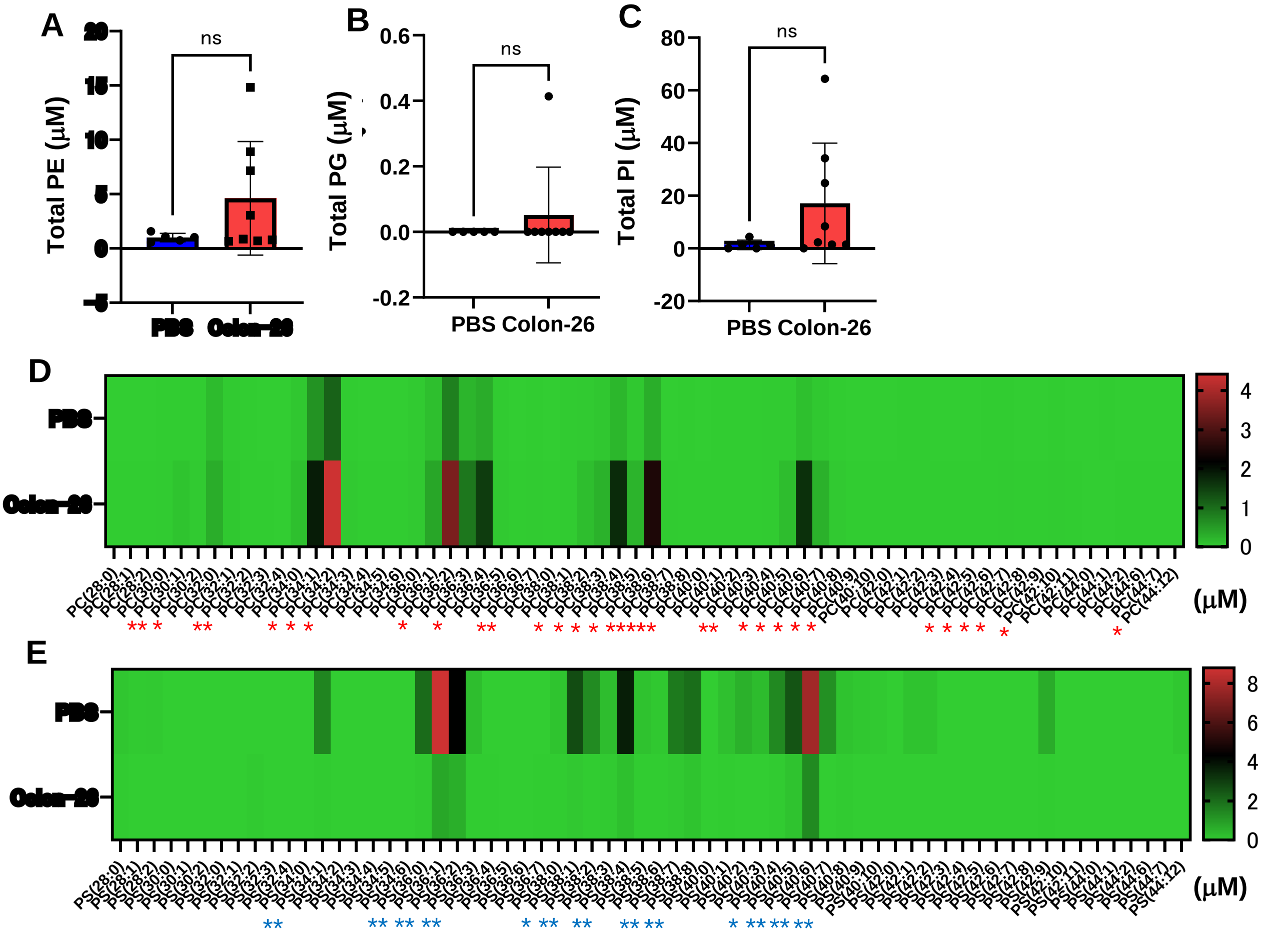

Supplemental Figure S4. Modulations of diacyl-phospholipids in the ascitic fluid of murine models of carcinomatous peritonitis

Modulations of total PE (A), total PG (B), total PI (C), PC species (D), and PS species (E) in the ascitic fluid, illustrated in Figure 1, are shown. Differences were evaluated using the Mann-Whitney U test. **P* < 0.05, ***P* < 0.01. The boxes represent the means of independent samples, and the bars represent the S.D. The modulations of PC and PS species are shown as heat maps.

Supplemental Figure S5

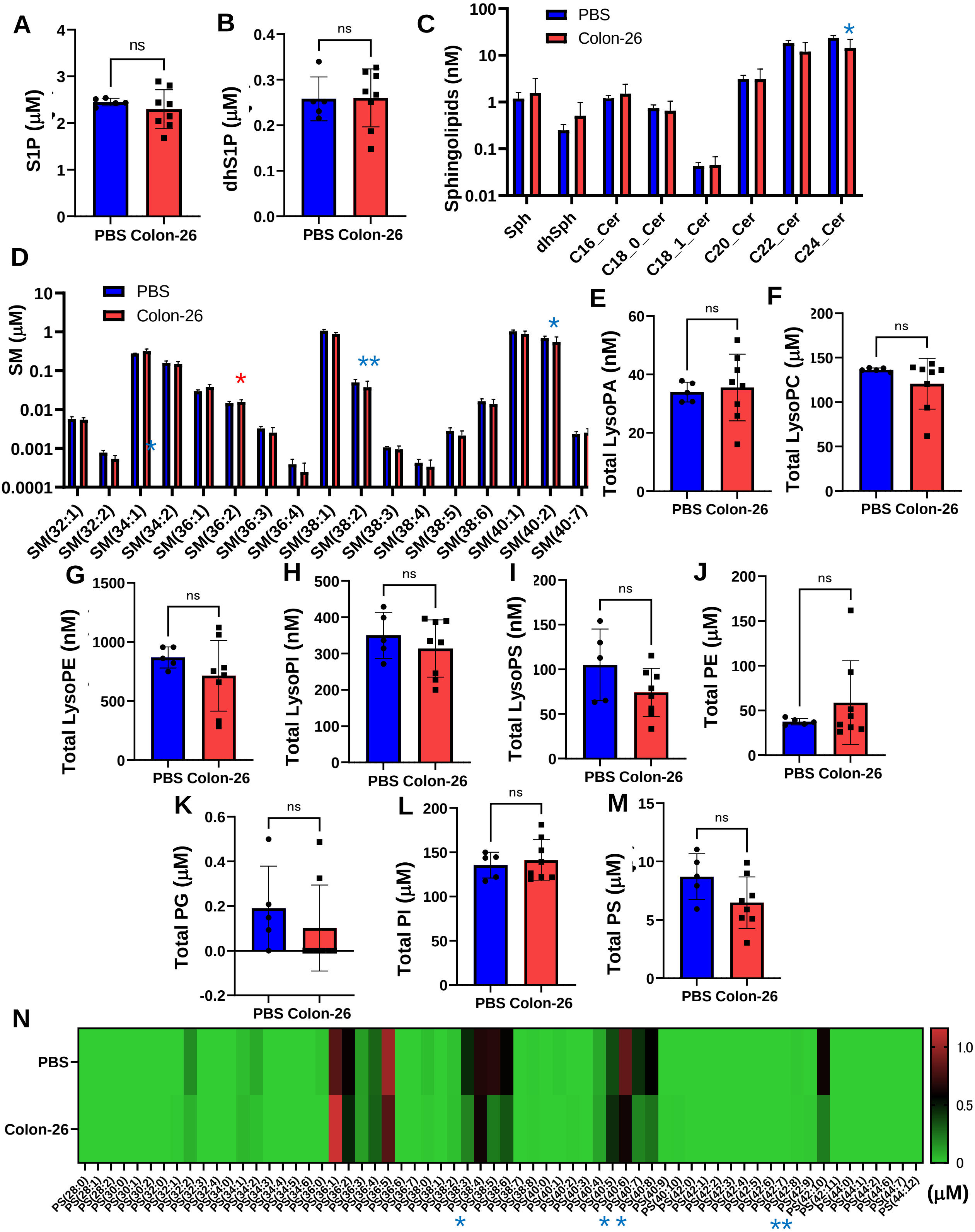

Supplemental Figure S5. Modulations of sphingolipids, lysophospholipids, and diacyl-phospholipids in the plasma of murine models of carcinomatous peritonitis

Modulations of S1P (A), dhS1P (B), sphingolipids (C), SM species (D), total LysoPA (E), total LysoPC (F), total LysoPE (G), total LysoPI (H), total LysoPS (I), total PE (J), total PG (K), total PI (L), total PS (M), and PS species (N) in the plasma, illustrated in Figure 3, are shown. Differences were evaluated using the Mann-Whitney U test. **P* < 0.05, ***P* < 0.01. The boxes represent the means of independent samples, and the bars represent the S.D. The modulation of PS species is shown as a heat map.

Supplemental Figure S6

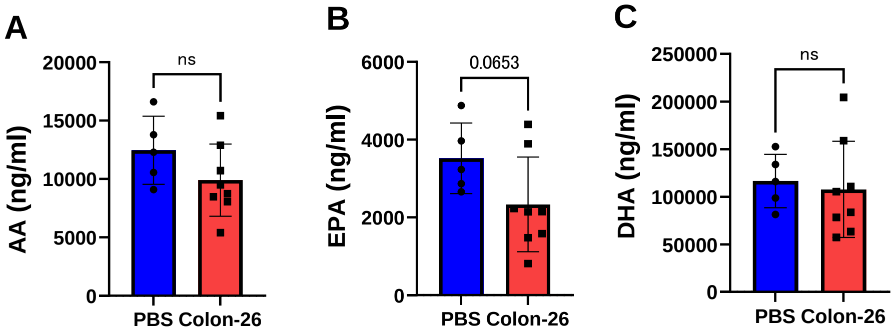

Supplemental Figure S6. Modulations of fatty acids in the plasma of murine models of carcinomatous peritonitis

Modulations of AA (A), EPA (B), and DHA (C) in the plasma, illustrated in Figure 3, are shown. Differences were evaluated using the Mann-Whitney U test. $*P < 0.05$, $**P < 0.01$. The boxes represent the means of independent samples, and the bars represent the S.D.

Supplemental Figure S7

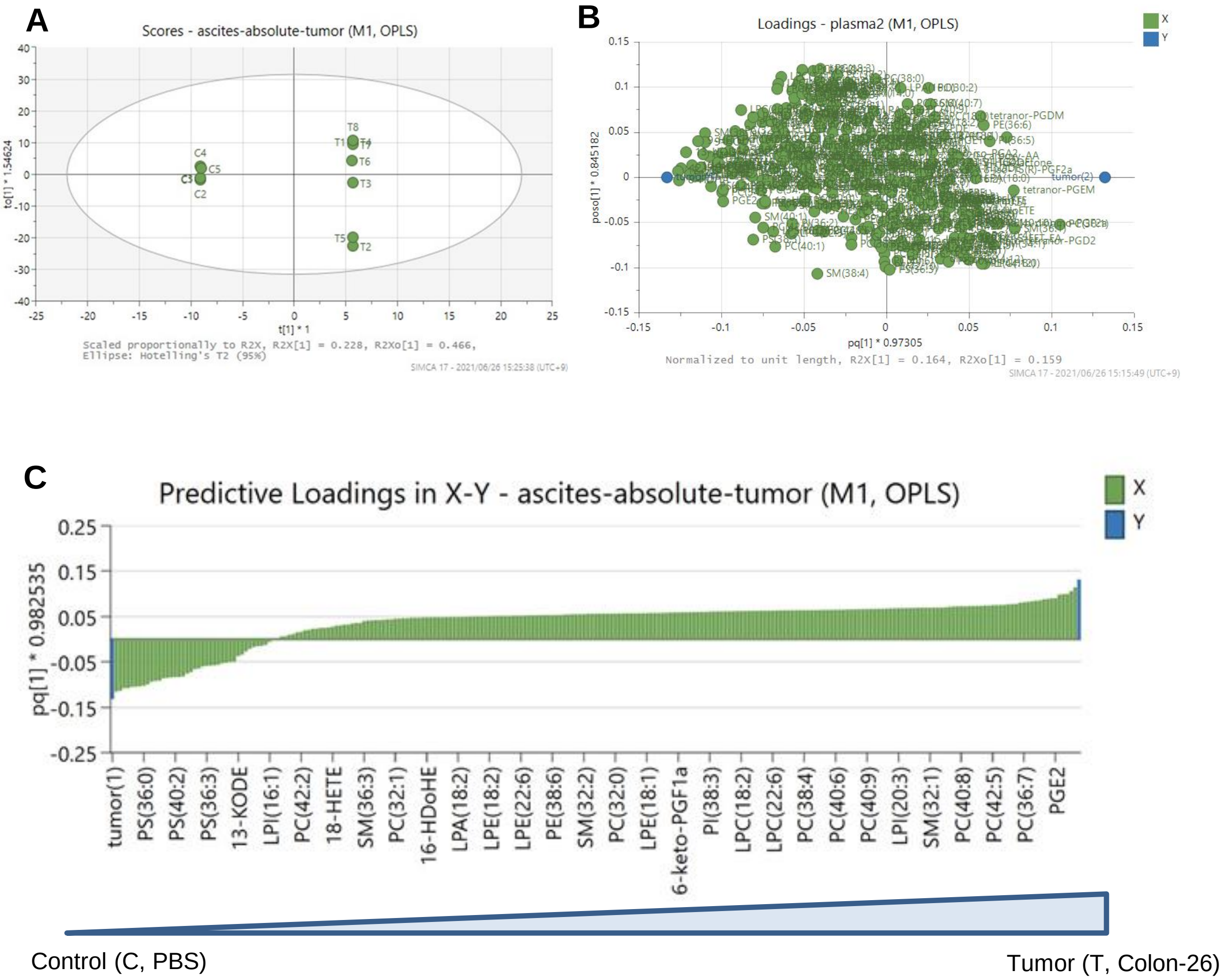

Supplemental Figure S7. OPLS analysis for the simultaneously measured lipid mediators in the ascitic fluid with variable importance in projection for carcinomatous peritonitis.

An OPLS analysis was performed to investigate all the measured lipid mediators in the ascitic fluid with variable importance in projection for carcinomatous peritonitis. The score plot (A), loading scatter plot (B), and loading column plot (C) for the OPLS are shown.

Supplemental Figure S8

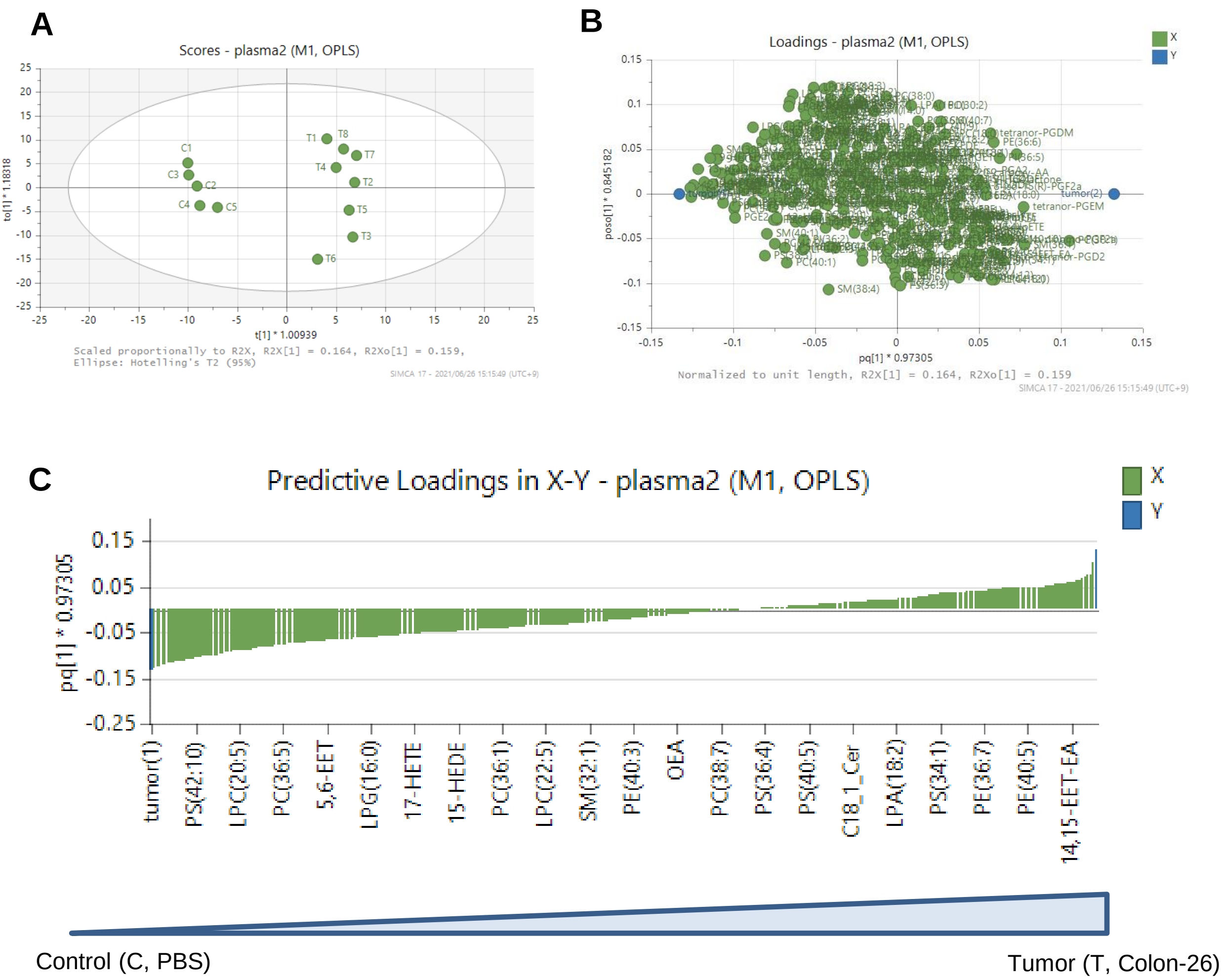

Supplemental Figure S8. OPLS analysis for the simultaneously measured lipid mediators in the plasma with variable importance in projection for carcinomatous peritonitis.

An OPLS analysis was performed to investigate all the measured lipid mediators in the plasma with variable importance in projection for carcinomatous peritonitis. The score plot (A), loading scatter plot (B), and loading column plot (C) for the OPLS are shown.

Supplemental Figure S9

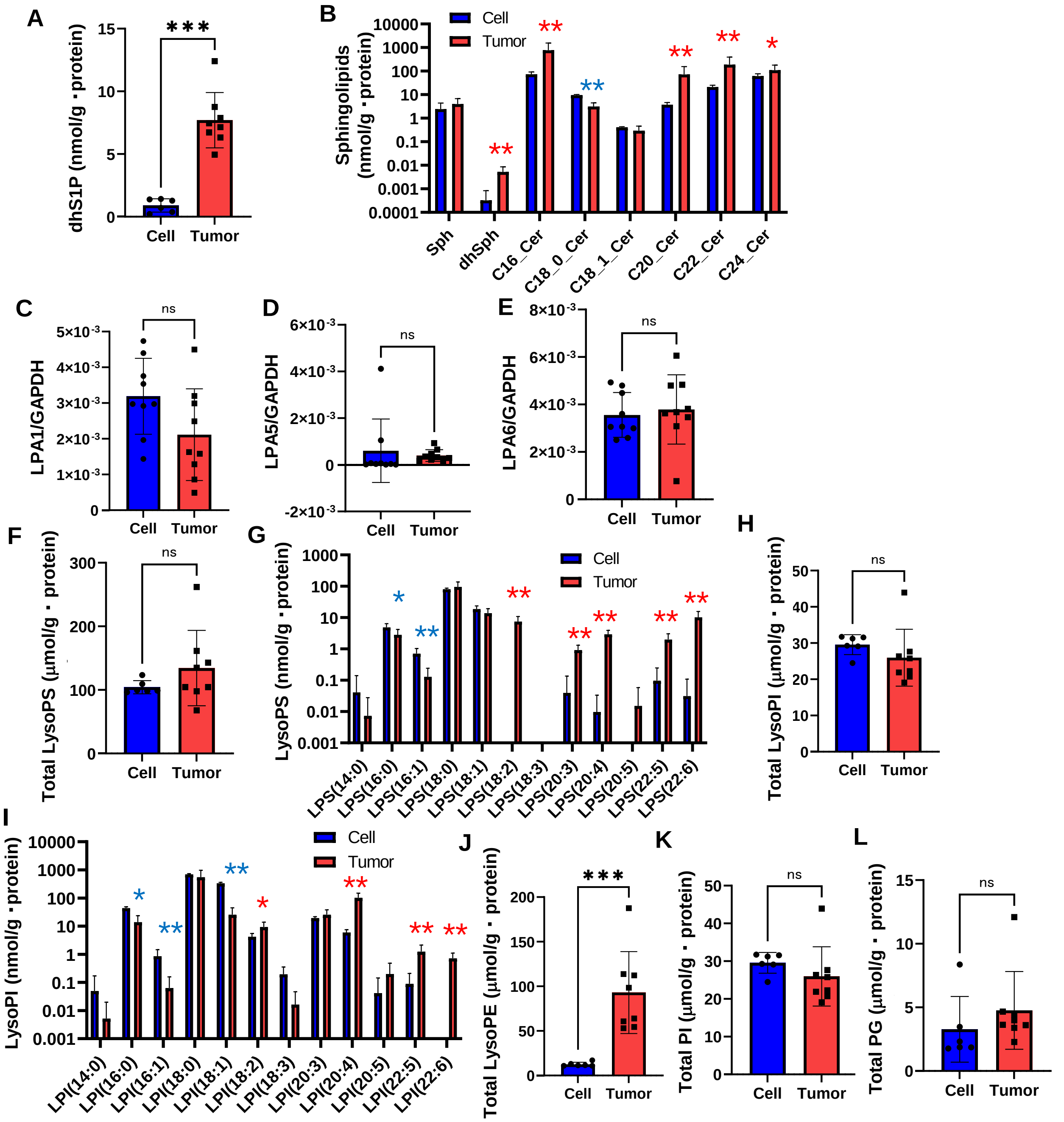

Supplemental Figure S9. Modulations of the expressions of the receptors for LPA, and the contents of sphingolipids, lysophospholipids, and diacyl-phospholipids within the Colon-26 tumor xenograft tissue

Colon-26 cells were seeded on to 6-well plates at the same time, and Colon-26 cells from some wells were injected intraperitoneally into Balb/c mice, as illustrated in Figure 1 to Figure 3, and those from other wells were collected for analyses (n = 6). The cancerous tissues from the Colon-26 tumor xenografts were collected for further analyses (n = 8). The mRNA expression levels of the receptors for lipid mediators were determined by a real-time PCR method using GAPDH as the internal standard. The lipid contents were determined by LC-MS/MS. (A) dhS1P, (B) sphingolipids, (C–E) mRNA expression levels of LPA receptors, (F) total LysoPS, (G) LysoPS species, (H) total LysoPI, (I) LysoPI species, (J) total LysoPI, (K) total PI, and (L) total PG. Differences were evaluated using the Mann-Whitney U test. *$P < 0.05$, **$P < 0.01$, ***$P < 0.001$, ****$P < 0.0001$. The boxes represent the means of independent samples, and the bars represent the S.D.

Supplemental Figure S10

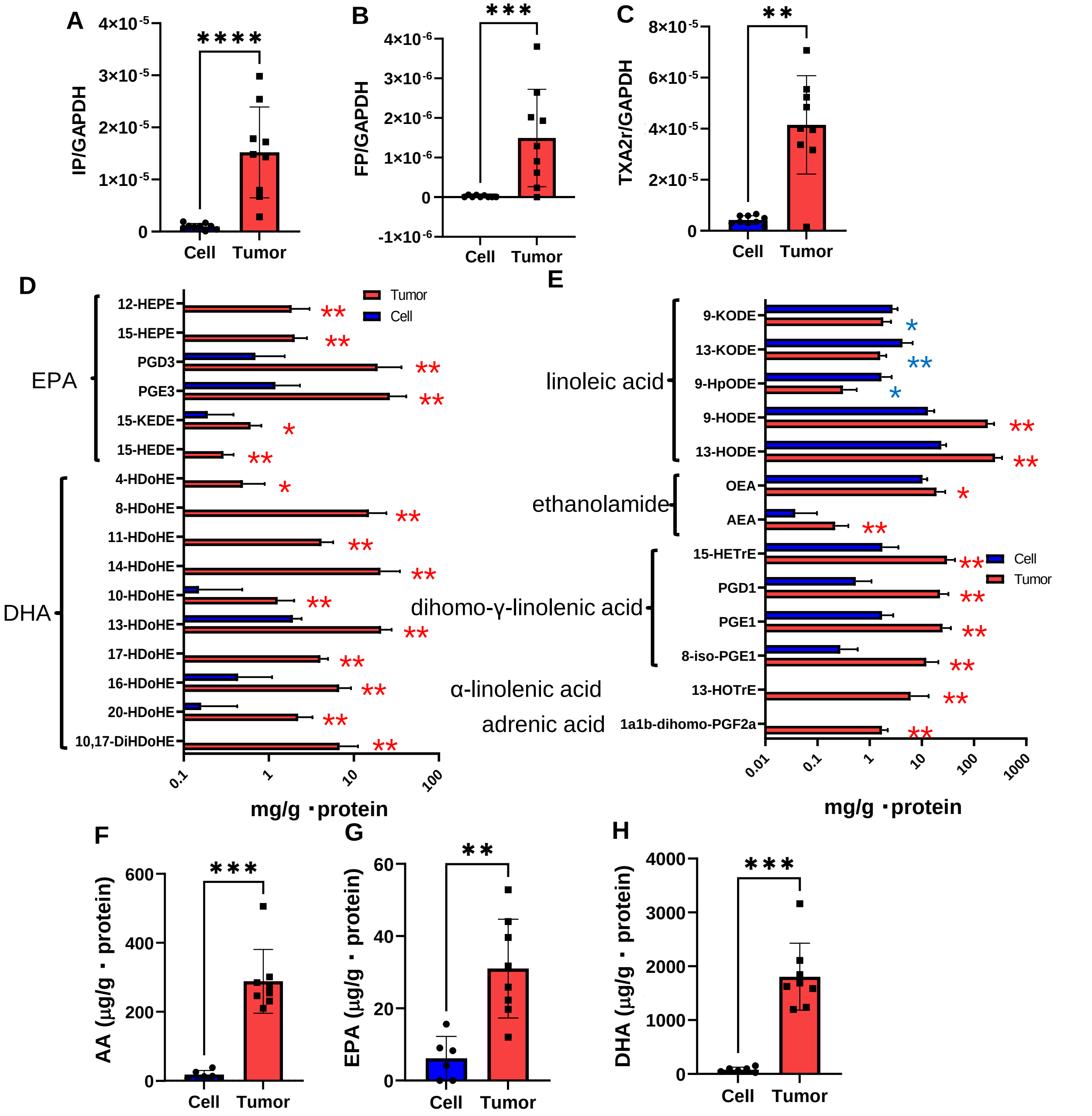

Supplemental Figure S10. Modulations of the expressions of the receptors for eicosanoids, and the contents of eicosanoids and related mediators in the cancer cells within the Colon-26 tumor xenograft tissue

Colon-26 cells were seeded on to 6-well plates at the same time, and Colon-26 cells from some wells were injected intraperitoneally into Balb/c mice, as illustrated in Figure 1 to Figure 3, and those from other wells were collected for analyses (n = 6). Specimens of cancerous tissues from the Colon-26 tumor xenografts were collected for further analyses (n = 8). The mRNA expression levels of the receptors for lipid mediators were determined by a real-time PCR method using GAPDH as the internal standard. The lipid contents were determined by LC-MS/MS. (A–C) mRNA expression levels of the eicosanoid receptors, (D) DHA and EPA derivatives, (E) other eicosanoid-related mediators, and (F) AA, (G) EPA, and (H) DHA. Differences were evaluated using the Mann-Whitney U test. $*P < 0.05$, $**P < 0.01$, $***P < 0.001$, $****P < 0.0001$. The boxes represent the means of independent samples, and the bars represent the S.D.
